# Biocatalytic Synthesis Using Self‐Assembled Polymeric Nano‐ and Microreactors

**DOI:** 10.1002/anie.202213974

**Published:** 2022-11-17

**Authors:** Yangxin Wang, Qingcai Zhao, Rainer Haag, Changzhu Wu

**Affiliations:** ^1^ College of Materials Science and Engineering Nanjing Tech University Puzhu Road(S) 30 211816 Nanjing P.R. China; ^2^ Institute of Chemistry and Biochemistry Freie Universität Berlin Takustrasse 3 14195 Berlin Germany; ^3^ Department of Physics Chemistry and Pharmacy University of Southern Denmark Campusvej 55 5230 Odense Denmark; ^4^ Danish Institute for Advanced Study University of Southern Denmark Campusvej 55 5230 Odense Denmark

**Keywords:** Biocatalytic Synthesis, Enzymatic Reactions, Polymeric Reactors, Self-Assembly

## Abstract

Biocatalysis is increasingly being explored for the sustainable development of green industry. Though enzymes show great industrial potential with their high efficiency, specificity, and selectivity, they suffer from poor usability and stability under abiological conditions. To solve these problems, researchers have fabricated nano‐ and micro‐sized biocatalytic reactors based on the self‐assembly of various polymers, leading to highly stable, functional, and reusable biocatalytic systems. This Review highlights recent progress in self‐assembled polymeric nano‐ and microreactors for biocatalytic synthesis, including polymersomes, reverse micelles, polymer emulsions, Pickering emulsions, and static emulsions. We categorize these reactors into monophasic and biphasic systems and discuss their structural characteristics and latest successes with representative examples. We also consider the challenges and potential solutions associated with the future development of this field.

## Graphical abstract

The use of polymeric systems for bioconversion is an efficient and recyclable technology for the future industry due to its zero‐pollution features. This Review focuses on self‐assembled polymeric nano‐ and micro‐sized structures and their application as biocatalytic reactors for chemical synthesis, including polymersomes, reverse micelles, polymer emulsions, Pickering emulsions, and static emulsions.

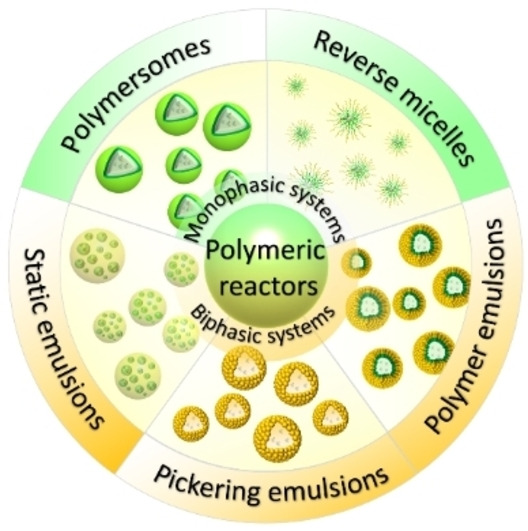

## 1 Introduction

In nature, biocatalysis may take place in compartmentalized cellular environments surrounded by membranes self‐assembled from amphiphilic molecules, allowing multiple spatially separated chemical transformations to produce complex compounds with high specificity and accuracy. With the rapid expansion of the range of available enzymes and chemical reactions, biocatalysis is becoming an important tool for organic synthesis, holding great potential in the modern chemical and pharmaceutical industries.^[1]^ However, enzymes tend to malfunction when they are used out of cellular environments. To solve this problem, researchers have devoted considerable efforts to creating biocatalytic systems with reactors comprised of artificial self‐assembling nano‐ and microscale compartments possessing a solvent‐filled volume partitioned from the surrounding environment. These nano‐ and microscale compartments can offer protection for the encapsulated enzymes from abiological conditions.^[2]^ Meanwhile, the large surface area of these nano‐ or microscale compartments also contributes to a higher reaction rate compared to bulk materials with immobilized enzymes. Besides, the biocatalytic reactions can occur with higher selectivity or fewer side reactions within the confined space.^[3]^ To date, a large variety of molecules and materials have been used to construct nano‐ and microscale self‐assembling reactors for chemical synthesis, such as small molecular surfactants,^[4]^ metal oxides,^[5]^ SiO_2_,^[6]^ metal–organic frameworks (MOFs),^[7]^ and polymers.^[8]^ Among these, polymers, with their tunable self‐assembly behavior, are considered excellent candidates for making such biocatalytic reactors because they offer a few key benefits over other options. Unlike small molecular surfactants, the use of polymers benefits from their easy recycling, for example, by filtration or solvent precipitation.^[9]^ Furthermore, the wide portfolio of available monomers offers a range of effective tools for constructing a rich array of polymers to form polymeric nano‐ and microscale reactors. Such reactors can be designed with different reactive functional groups, allowing easy pre‐ and post‐modification to endow the reactors with more practical functions, higher stability, and optimal permeability.^[10]^ More importantly, these polymeric reactors can be built with biocompatible and biodegradable polymers, allowing for environmentally friendly biocatalysis.^[11]^

**Figure 1:**
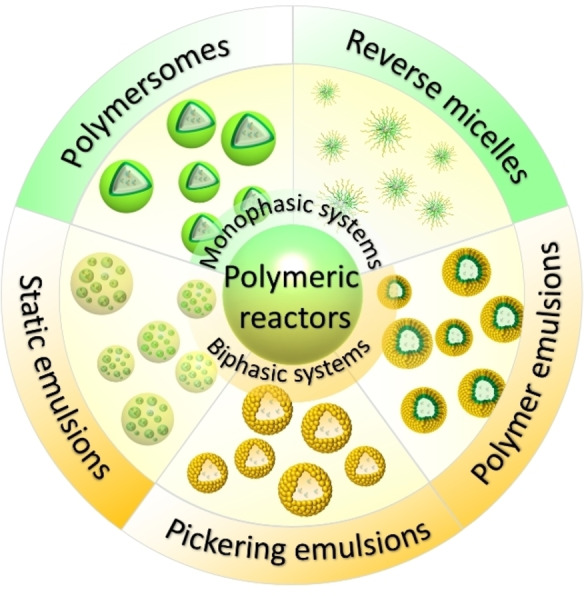
Representative illustration of self‐assembling polymeric reactors described in the present Review.

Based on their structural characteristics and dispersion medium, most of the self‐assembling polymeric compartments involved in biocatalysis can be categorized into polymersomes, reverse micelles, layer‐by‐layer assemblies, and emulsions.^[12]^ Despite the multitude of possible structures, polymersomes, reverse micelles, and emulsions are the ones most widely used in biocatalytic synthesis (Figure 1). A polymersome is a spherical, hollow self‐assembly of amphiphilic polymers in aqueous media with an inner aqueous compartment, while reverse micelles are obtained from the self‐assembly of amphiphilic polymers in an organic solvent. Distinctively, polymeric emulsions are formed in biphasic solutions, which are favorable for dissolving biocatalysts, substrates, and products in the ideal solvents. These polymeric reactors, applied to biocatalytic reactions, usually simplify the separation of products from the reaction system, sidestepping the problems associated with small molecular surfactants. Additionally, polymers are often inert materials that exert no negative influence on the vulnerable biocatalysts, helping instead to retain their activity against the hazardous environment outside of the compartment.^[13]^ Furthermore, polymeric reactors constructed through self‐assembly can be designed to be easily recyclable and controllable, offering functionality such as switching catalytic reactions on and off and separating products on demand. With this array of advantages, self‐assembling polymeric reactors provide enormous opportunities for efficient biocatalysis.

The past decades have seen plentiful achievements in the area of polymeric biocatalytic reactors, and several excellent review articles have been published. For instance, the Palivan group contributes a review article about enzymatic reactions performed in polymeric compartments, focusing mainly on preparation techniques, biomedical applications, and artificial cell mimics.^[14]^ The Cuomo group's review article covers various aspects of the use of polymeric capsules for biocatalysis, including fabrication routes, responsiveness, and mass transfer phenomena, with an emphasis on biocatalytic events with high potential for application in various fields.^[12b]^ However, self‐assembled polymeric nano‐ and microcapsules for enzymatic synthesis are only briefly mentioned in the above reviews. Importantly, even though chemical synthesis is enzymes’ key application in both industry and academia, no overview to date has focused on self‐assembled polymeric structures as biocatalytic reactors for synthetic purposes. Considering our recent achievements in polymeric emulsions for biotransformations, as well as the growing number of publications on self‐assembled polymeric reactors for biocatalytic synthesis, the present Review seeks to provide an update on the most recent and significant advances in the relevant fields. In particular, it focuses on self‐assembled polymeric nano‐ and microscale structures in their application as biocatalytic reactors for chemical synthesis. The Review divides these reactors into two categories according to the reaction media, monophasic or biphasic, in which they operate. For both types of media, we aim on the one hand to showcase the diversity of self‐assembled nano‐ and microscale polymeric structures for synthetically useful biocatalytic reactions. On the other hand, each of the two topics is further divided into subsections based on the structural differences on display in these structures’ polymeric self‐assembly.

## 2 Polymeric Reactors in Monophasic Solution

Biocatalytic reactions in monophasic solution mainly refer to reactions carried out in either an aqueous or an organic solution. Polymersomes^[15]^ and reverse micelles^[12c]^ are the most thoroughly investigated polymeric reactors for biocatalytic reactions in aqueous and organic media, respectively. Layer‐by‐layer assemblies of polymers have also been successfully used to encapsulate enzymes, but these assemblies are used mainly for biomedical applications other than biocatalytic synthesis, thus not discussed here.^[16]^

### 2.1 Polymersomes

A polymersome is a nanoscale, hollow, spherical capsule, self‐assembled in water from amphiphilic block copolymers, which contains an inner aqueous compartment.^[17]^ As compared to liposomes, the membranes of polymersomes are considerably thicker, and the interaction between these polymers is stronger due to the longer chains and higher functionality of polymers. These features enable polymersomes to protect the encapsulated biocatalysts from harsh reaction conditions, making polymersomes promising for encapsulating biocatalysts to form nanoscale reactors.^[18]^

The general range of polymers used for making polymersomes along with their physical properties, such as stability and permeability, have been well summarized in a previous review.^[19]^ Herein, we will only focus on the application of polymersomes as nanoreactors in this section. First of all, polymersomes’ stability is always important for their application as enzyme carriers during catalysis as well as long‐term storage.^[20]^ Crosslinking the copolymers that form the membranes was found to be an effective way to enhance the mechanical stability of polymersomes.^[21]^ In this strategy, due to the flexibility in the chemical design of the copolymers, polymerizable methacrylate groups are introduced as either end groups^[22]^ or side groups.^[23]^ After the formation of polymersomes, the copolymers can be crosslinked under mild conditions through photo‐initiated polymerization, maintaining the integrity and persistent shape of the polymersomes. In another example, the Nolte group reported the immobilization of biocatalyst‐loaded polymersomes in a hyaluronic acid hydrogel that can effectively enhance the stability and recyclability of the polymersome reactors.^[24]^ The hydrogel‐stabilized polymersomes containing the enzymes *Candida antarctica* lipase B (CalB) and glucose oxidase (GOx) were used to construct a ‘continuous‐flow reactor’, demonstrating the ability to process cascade reactions by converting 2‐methoxyphenyl acetate to tetraguaiacol together with chloroperoxidase from *Caldariomyces fumago* (CPO). This strategy requires that the gelation carried out in mild conditions should not influence the activity of the biocatalyst or the structural integrity of the polymersomes.

**Figure 2:**
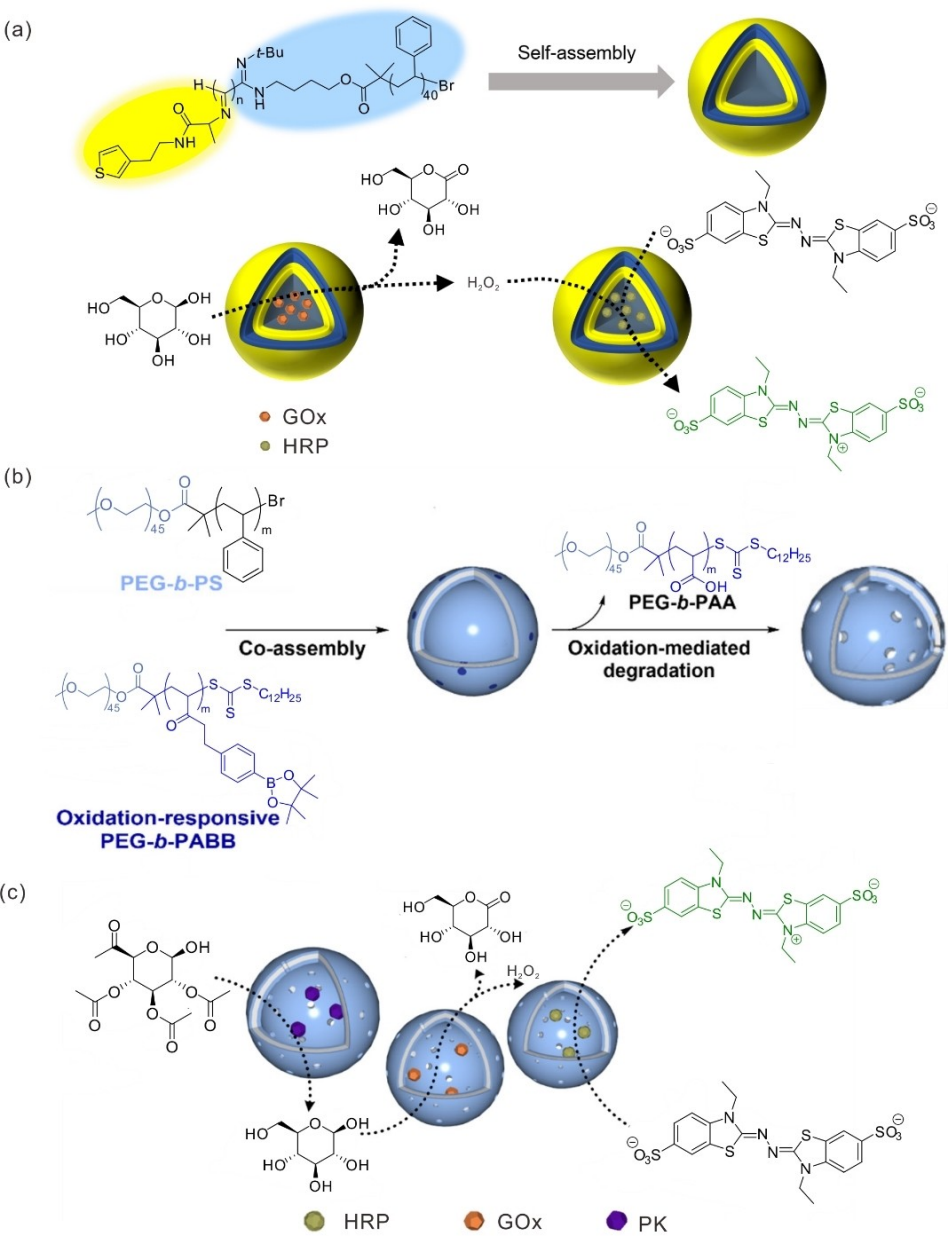
a) Chemical structure of PS–PIAT, which forms intrinsically porous polymersomes, and the schematic representation of a cascade reaction between the separate polymersomes containing different enzymes. b) Schematic depiction of the synthetic process of polymersomes with size‐selective permeability and c) the cascade reaction performed in these polymersomes using proteinase K (PK), GOx and HRP as biocatalysts. Adapted with permission from Ref. [31]. Copyright 2020 American Chemistry Society.

Permeability is another important factor that must be considered when using polymersomes as nanoreactors for biocatalytic reactions.^[25]^ Enhancing the permeability of polymersome membranes allows the fast passage of the substrates and products of enzymatic reactions, which is highly desirable when the system is used in organic synthesis. It is easy to understand that permeability is derived largely from the chemical composition and structures of the polymers forming the polymersomes.^[26]^ Poly(styrene)‐*b*‐polyisocyanoalanine(2‐thiophene‐3‐yl‐ethyl)amide (PS‐*b*‐PIAT), which has a rigid rod polyisocyanide head group and a flexible polystyrene tail, is capable of forming intrinsically porous polymersomes, resulting in reactors that allow the free diffusion of small molecular substrates while keeping large enzymes inside (Figure 2a).^[27]^ These polymersomes have been successfully used for cascade reactions by encapsulating GOx and horseradish peroxidase (HRP) separately in different polymersomes. Both GOx and HRP retained higher activity for longer periods in the PS–PIAT polymersomes than free ones. Channel‐equipped polymersomes have also attracted much research attention because channels endow polymersomes with good permeability to maintain sufficient mass transfer and thus efficient catalysis. In this strategy, membrane proteins^[28]^ and DNA nanopores^[29]^ are incorporated into membranes as size‐selective channels that allow only the passage of small molecules while confining the larger enzymes. The Castiglione group^[28e]^ demonstrated that different membrane proteins can be inserted into the same polymer membranes of polymersomes, alleviating the mass transfer limitations of chemically diverse molecules. In these polymersomes, which contained two kinds of enzymes—ketoreductase and formate dehydrogenase—the membrane proteins AlkL, OmpW, OprG, and TodX were investigated for transporting the substrates and products of ketoreductase, while OmpF, PhoE, and FocA were studied for the transport of formate. The highest channel‐specific effects on mass transfer of the polymersomes were achieved with TodX and PhoE, which led to an improvement of the space–time yield of the final product (*S*)‐pentafluorophenyl ethanol by 2.32‐fold compared to polymersomes without membrane proteins. In any case, the successful incorporation of these components into catalytically active polymersomes represents a new step to reduce mass transport limitations, although the high expense and difficulty of handling membrane proteins and DNA nanopores constitute significant challenges. But simple and economical methods do exist for constructing polymersomes with high permeability toward small molecules, offering a great opportunity to enhance catalytic activity. Because of the pH‐ and sugar‐responsive disassembly behavior of poly(ethylene glycol)‐*b*‐poly(styrene boronic acid) (PEG‐*b*‐PSBA), PEG‐*b*‐PSBA in polymersomes dissolves in the presence of sugar or OH^−^, allowing for the creation of a highly permeable membrane.^[30]^ After encapsulating CalB, hydrolysis of 6,8‐difluoro‐4‐methylumbelliferyl octanoate (DiFMU octanoate) and *p*‐nitrophenyl acetate was carried out in the semipermeable nanoreactors, while no activity was observed before the disassembly of PEG‐*b*‐PSBA. A similar strategy for controlling the permeability of polymersomes was reported by the Kim group.^[31]^ Their polymersomes were constructed through the self‐assembly of poly(ethylene glycol)‐*b*‐polystyrene (PEG‐*b*‐PS) and poly(ethylene glycol)‐*b*‐poly(acrylbenzylborate) (PEG‐*b*‐PABB). The benzyl borate pendants of PABB can be oxidized in the presence of H_2_O_2_ to poly(acrylic acid) (PAA). Due to the dissolution of PEG‐*b*‐PAA, this system achieved size‐selective permeability that allowed the passage of substrates and products while confining the biocatalysts within the polymersomes and maintaining structural integrity (Figures 2b and c). But such a strategy to produce semipermeable polymersomes requires the design of copolymers that can form phase‐separated domains in polymer membranes, which become voids after being treated with specific stimuli. Meanwhile, it should be noted that the permeability change of polymersomes controlled by this method is not reversible. The Bruns group reported a general method for generating semipermeable nanoreactors through the photoreaction of 2‐hydroxy‐4′‐2‐(hydroxyethoxy)‐2‐methylpropiophenone (PP−OH) with a membrane of polymersomes self‐assembled from different amphiphilic block copolymers, causing a chemical modification of the polymer membranes with hydrophilic PP−OH to enhance their permeability toward small molecules.^[32]^ With entrapped enzymes, expected products were generated when the biocatalytic reactions were carried out in such polymersomes after photoreaction treatment, while almost no product was detected without the treatment. It is worth mentioning that this photoreaction treatment remains viable irrespective of the chemical structures of the polymers that form the polymersomes.

**Figure 3:**
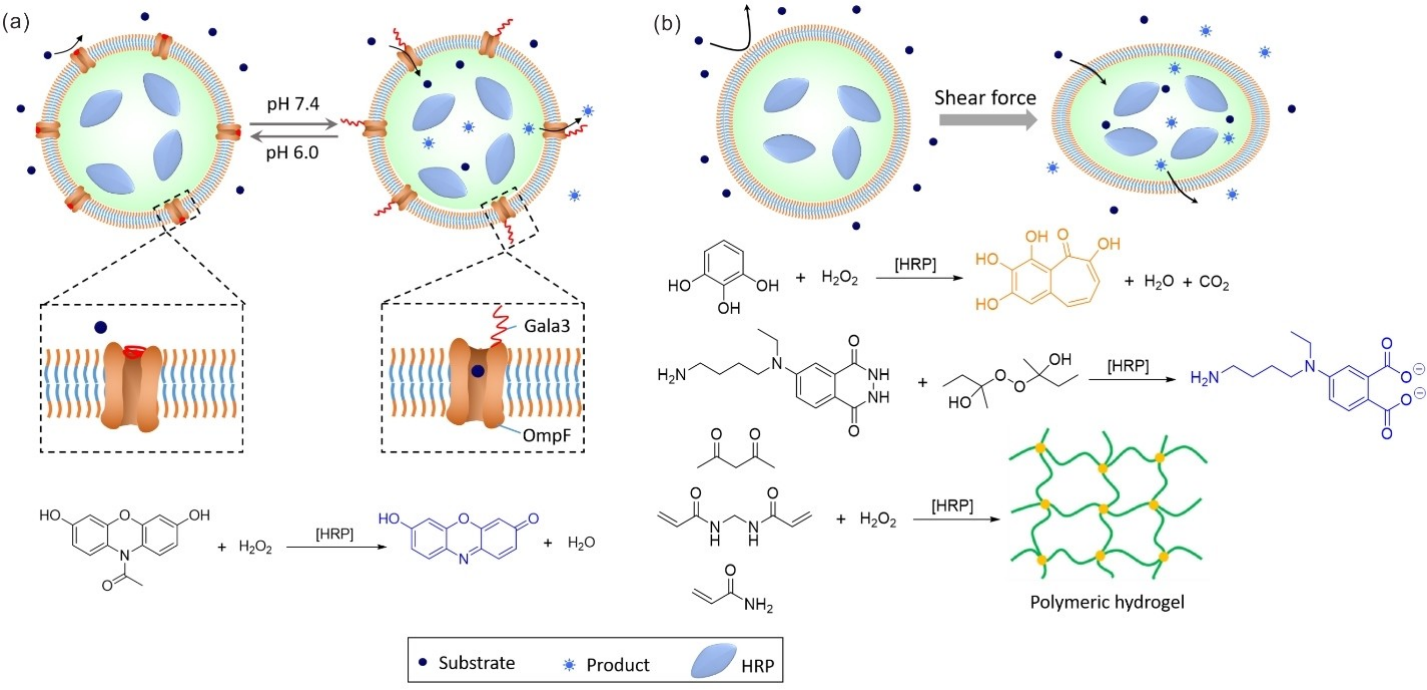
a) Schematic representation of a biovalve functioning by reversible pore opening and closing inside the membrane of polymersomes to trigger an *in situ* reaction (left: closed state; right: open state). The OmpF pore modified with Gala3 is inserted into the polymersome membrane that separates the encapsulated enzyme (HRP) from the environment. The *in situ* reaction is triggered by the biovalve functionality, which allows diffusion through the OmpF pores of the substrate Amplex UltraRed and the subsequent release of the fluorescent products. b) Schematic depictions of the enzymatic reaction performed in a shear‐responsive polymersome reactor, and schemes of different catalytic reactions carried out in such stimuli‐responsive polymersomes containing HRP.

Responsive polymersomes, whose permeability can be reversibly modulated by external stimuli, are of great interest for operating biocatalytic reactions in a controlled manner so as to switch the reactions on and off in an on‐demand fashion. The pH‐sensitive reactor is the most popular responsive polymersome system. In these polymersomes, polymers show different conformations and/or configurations under different pH conditions, so polymersome membranes’ permeability towards substrates can be modulated by tuning pH values, thus halting or initiating the catalytic reaction. To construct such polymersomes, amphiphilic block polymers must contain a suitable number of pH‐sensitive segments, either in the hydrophilic part^[33]^ or the hydrophobic part,[^23^, ^34^] and the encapsulated biocatalysts should maintain their catalytic activity throughout the range of varying pH values. Another strategy for building pH‐responsive polymersome reactors is incorporating responsive channels into polymersomes. In these polymersomes, only the channels are sensitive to pH changes. For example, by the insertion of pH‐responsive biovalves, which was achieved by attaching stimuli‐responsive peptide sequence LAEALAEALAEA (Gala3) to a genetically modified channel porin OmpF, Palivan and colleagues developed catalytic reactors containing HRP whose *in situ* activity could be switched on and off when pH was changed between 7.4 and 6.0, respectively (Figure 3a).^[35]^ As it was revealed that the free HRP lost its activity very fast under these cycling pH changes, the pH‐responsive OmpF‐equipped polymersomes were proven to offer sufficient protection to the encapsulated enzyme as it could maintain its activity after several cyclings of pH changes. The Bruns group reported a kind of shear stress‐responsive polymersome nanoreactor, inspired by the marine bioluminescence of dinoflagellates, whose membrane can be transiently switched between impermeable and semipermeable states by the shear forces occurring in flow or under turbulent mixing (Figure 3b).^[36]^ The applied shear force made nucleobase pairs in the hydrophobic leaflet in the membrane separate, exposing the hydrogen bonding motifs and making the membrane less hydrophobic and more permeable for water‐soluble compounds. HRP was encapsulated in such polymersomes, and switchable catalytic activity was demonstrated through various reactions, including the catalytic conversion of pyrogallol to purpurogallin, the luminescence reaction of *N*‐(4‐aminobutyl)‐*N*‐ethylisoluminol, and the free‐radical polymerization of vinyl monomers.

**Figure 4:**
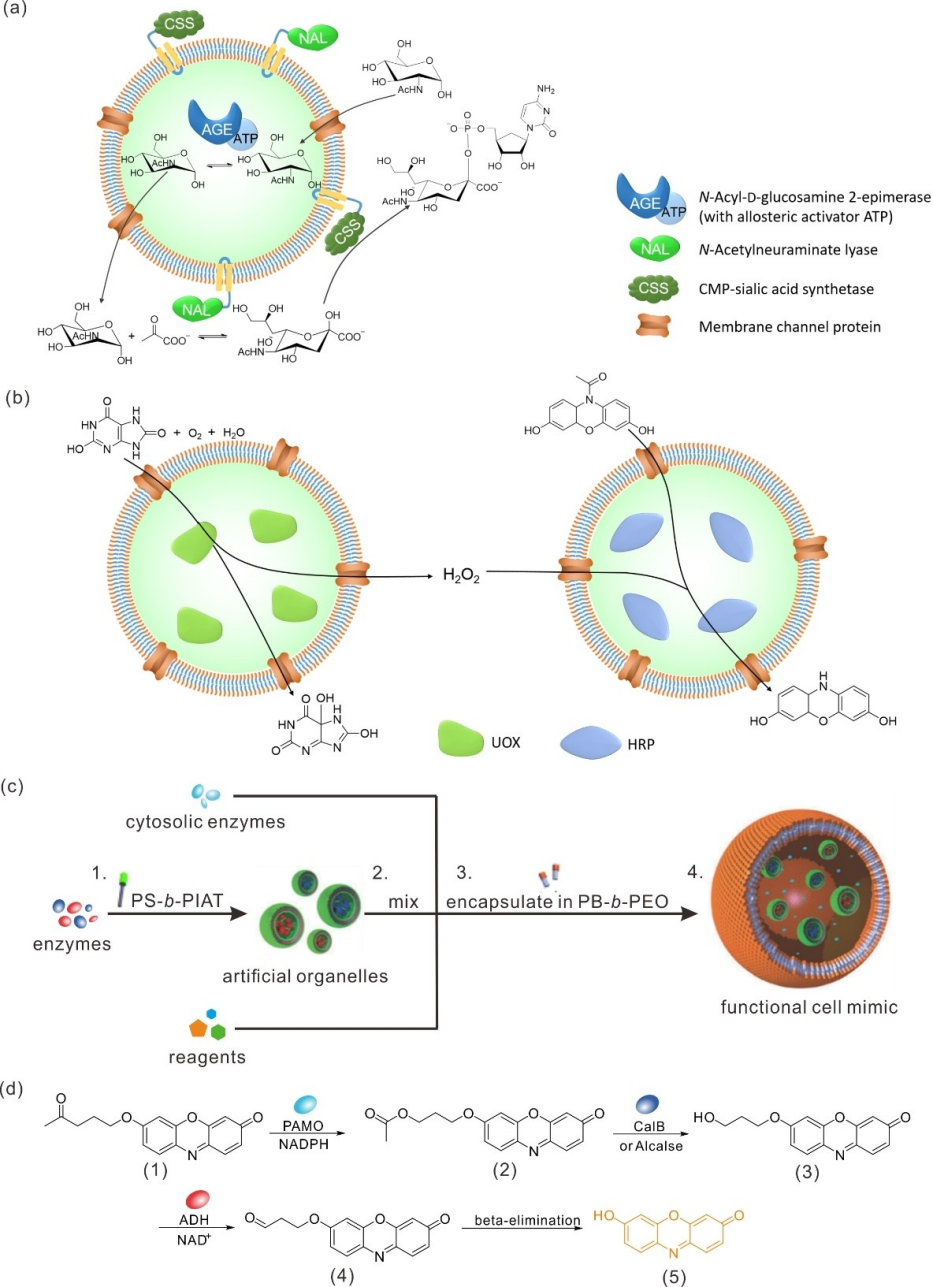
a) Schematic depiction of three‐step synthesis of CMP–*N*‐acetylneuraminic acid in channel protein‐equipped polymersomes containing three different enzymes separated spatially. b) Schematic depiction of biocatalytic cascade reactions performed in spatially segregated polymersomes. c) Schematic depiction of the formation of a multi‐compartmentalized polymersome (vesosome) as a functional cell mimic. It shows the initial encapsulation of different enzymes in PS‐*b*‐PIAT sub‐compartments (1), followed by the mixing of the sub‐compartments, cytosolic enzymes and reagents (2), and the encapsulation of the reaction mixture in a PS‐*b‐*PEO main compartment (3), to form the final multi‐compartmentalized polymersome as a functional cell mimic (4). d) Schematic illustration of the four‐step cascade reaction performed in the multi‐compartmentalized polymersomes. Adapted from Ref. [39] with permission from Wiley‐VCH.

Following the success of the single enzymatic reaction, two‐step[^27^, ^28e^, ^28f^, ^37^] and three‐step[^31^, ^38^] biocatalytic cascade reactions were successfully carried out in polymersome reactors. Enzymes can be not only positioned in the water pool of polymersomes, but also entrapped in the membranes or even anchored to the external surface of polymersomes, thus allowing the spatial localization of different enzymes within one single polymersome at different positions. For example, CalB, GOx and HRP were combined into one system for a three‐step cascade reaction. In this setup, GOx is always entrapped within the water pool of the polymersome, while the CalB and HRP are loaded in one of two ways: either HRP is embedded in the polymersomes’ lumen, with CalB tethered to the polymersome's surface,^[38a]^ or vice versa.^[38b]^ In either case, CalB first converts the substrate 1,2,3,4‐tetra‐*O*‐acetyl‐β‐glucopyranose (GAc_4_) into glucose, which is further catalyzed by GOx and HRP to convert 2,2′‐azinobis(3‐ethyl‐benzothiazoline‐6‐sulfonic acid) (ABTS) to ABTS⋅^+^. It was found that the encapsulated enzymes were 100‐fold more active than free ones when considering the effective enzyme concentration in total reaction volume, though the apparent substrate conversion was slower within the polymersome than for the free enzymes. A similar strategy was employed for a three‐step synthesis of cytidine monophosphate *N*‐acetylneuraminic acid (CMP–Neu5Ac) using *N*‐acyl‐D‐glucosamine‐2‐epimerase (AGE), *N*‐acetylneuraminate lyase (NAL), and CMP–sialic acid synthetase (CSS) as biocatalysts; during this reaction, AGE was encapsulated within the polymersome self‐assembled from poly(2‐methyloxazoline)_15_‐poly(dimethylsiloxane)_68_‐poly(2‐methyloxazoline)_15_ (PMOXA_15_‐PDMS_68_‐PMOXA_15_) while NAL and CSS were immobilized on its external surface (Figure 4a).^[38c]^ It was demonstrated that cross‐inhibitions in the enzymatic cascade reactions were suppressed due to both the spatial separation among the three enzymes and the existence of highly selective channel proteins in the polymersome membranes. Compared to the non‐compartmentalized reaction, the three‐step synthesis of CMP–*N*‐acetylneuraminic acid was improved 2.2‐fold. The spatial separation of enzymes is also achieved by encapsulating incompatible enzymes in segregated nanocompartments so that the individual reactions of the cascade reaction can take place under optimal conditions.^[31]^ In the Palivan group's report, uricase (UOX) and HRP, once loaded into spatially segregated nanocompartments, worked in tandem cascade reactions to generate Amplex UltraRed, proving the protective role of the polymersomes. This research also indicates the importance of a balance between the protective role of polymersomes and the permeation of small molecules for efficient cascade reactions (Figure 4b).^[28f]^ In nature, cells are generally not organized as single compartments but divided into multiple compartments. This structure, which allows separate components to be located in different compartments, enables position‐specific catalysis and therefore allows the use of otherwise incompatible biocatalysts and substrates. Mimicking such a hierarchical structure, polymersomes within polymersome architectures, or so‐called “polymer vesosomes”, were constructed for biocatalytic synthesis.[^28d^, ^39^] For example, PS‐*b*‐PIAT was used to form sub‐compartments that were then encapsulated in main compartments composed of polybutadiene‐*b*‐poly(ethylene oxide) (PB‐*b‐*PEO). By separately encapsulating cytosolic enzymes, alcohol dehydrogenase (ADH), phenylacetone monooxygenase (PAMO), and CalB, a functional cell mimic was successfully constructed, and a four‐step cascade reaction was carried out (Figure 4c and d).^[39]^

In addition to small organic molecules, polymers can also be synthesized through biocatalytic reactions in polymersomes. Cornelissen and colleagues reported the synthesis of oligomers from the monomers 8‐octanolactone and dodecalactone via ring‐opening polymerization inside PS‐*b‐*PIAT polymersomes containing CalB.^[40]^ In this setup, the CalB was located in different positions within the polymersomes, i.e. the inner aqueous pool or the bilayer polymeric membrane, and the location of CalB exhibited a clear impact on the polymerization activity. The enzyme entrapped in the water pool showed similar reactivity as the free enzyme in an aqueous solution, while the enzyme located in the bilayer was sterically less accessible and produced shorter oligoester fragments. HRP encapsulated in polymersomes was reported to produce polymers with vinyl monomers because of its ability to generate free radicals in the presence of hydrogen peroxide.[^32b^, ^36^] For example, the atom transfer radical polymerization (ATRP) of poly(ethylene glycol) methyl ether acrylate catalyzed by HRP in the water pool of polymersomes was conducted, achieving polymer‐filled polymersomes constructed from hydrophilic polymers.^[32b]^ The successful preparation of intact polymersomes containing filled polymers is very important as they can function respectively as mimics of a cellular membrane and a viscous cytosol.

To summarize, the polymersome is among the most widely investigated polymeric reactors for biocatalysis. Because they are usually formed in an aqueous solution, polymersomes are favorable for the operation of enzymes, and capable of hosting biocatalytic reactions that yield not only small molecules but also polymers. Obviously, the polymersome‐based nanoreactors for biocatalytic synthesis exhibit several advantages over biocatalysis performed in an aqueous solution using free enzymes. First of all, polymersome can offer protection for the encapsulated biocatalysts from unsuitable reaction conditions. Secondly, controllable initiating and halting of reactions can be achieved when stimuli‐responsive polymersomes are used as the biocatalytic nanoreactors. Additionally, spatially separated localization of biocatalysts in polymersomes is beneficial to suppress the cross‐inhibition effect of different enzymes and maintain them working in their preferable conditions, which is favorable for biocatalytic cascade reactions. On the other hand, challenges still exist when applying polymersomes as nanoreactors for biocatalytic synthesis, such as poor solubility and low permeability of organic substrates, low encapsulation ratio of biocatalysts, and difficulty in large‐scale production of polymersome nanoreactors. Therefore, more investment is still needed for achieving the practical application of polymersomes in biocatalytic synthesis of valuable chemicals.

### 2.2 Polymeric Reverse Micelles

Although water is the solvent for enzymatic reactions taking place in nature, it is a rather poor solvent for nonpolar substrates. Transferring biocatalysis from an aqueous to an organic environment improves the availability of substrate in the organic solvent. However, many enzymes will be denatured or deactivated when exposed to organic solvents or concentrated organic substrates if not properly handled.^[41]^ The stability of enzymes is highly related to the polarity of organic solvents, which can be expressed by Log*P* (log[$\frac{C_{octanol}}{C_{water}}$ ]), where *C*_octanol_/*C*_water_ is the partition coefficient of a compound between *n*‐octanol and water. According to Laane's Rule, enzymes in nonaqueous solutions are generally more active in nonpolar solvents (log*P*>4) and less active in polar solvents (log*P*<2).^[42]^ On the other hand, a minimal amount of water in organic solvent is favorable for the stability of enzymes because it may keep the conformational mobility of enzymes at a suitable level.^[13a]^ To obtain biocatalysts that are stable in organic media, different strategies have been developed, including immobilizing enzymes on or in an inert matrix,^[43]^ formation of water‐insoluble enzyme particles,^[44]^ chemical modification of enzymes,^[45]^ stabilizing enzymes with additives^[46]^ or in reverse micelles.^[47]^ Reverse micelles, also called microemulsions, are optically isotropic colloidal dispersions of droplets of water in oil with diameters less than 10 nm, stabilized by self‐assembled surfactants. Because of the very small amount of water in the hydrophilic internal cavity, reverse micelles are classified as reactors in single‐phase organic media.^[48]^ Their combination of a hydrophobic exterior and a hydrophilic internal cavity shelters encapsulated enzymes from detrimental organic solvents.^[49]^ In another advantage of these systems, certain enzymes—for example, chymotrypsin and HRP—show superactivity behavior in some reverse micellar reaction systems, though there is still no clear and general understanding of the origin of such superactivity.^[50]^ However, due to the strong electrostatic and hydrophobic interactions between surfactants and biocatalysts, some reverse micellar reaction systems fabricated from small molecular surfactants exhibit reduced biocatalyst activity and stability.^[51]^ The high concentration of small molecular surfactants also makes product separation and enzyme recovery extremely laborious. For these reasons, amphiphilic polymers are recognized as good replacements for small molecular surfactants to construct reverse micelles for biocatalysis.^[52]^

**Figure 5:**
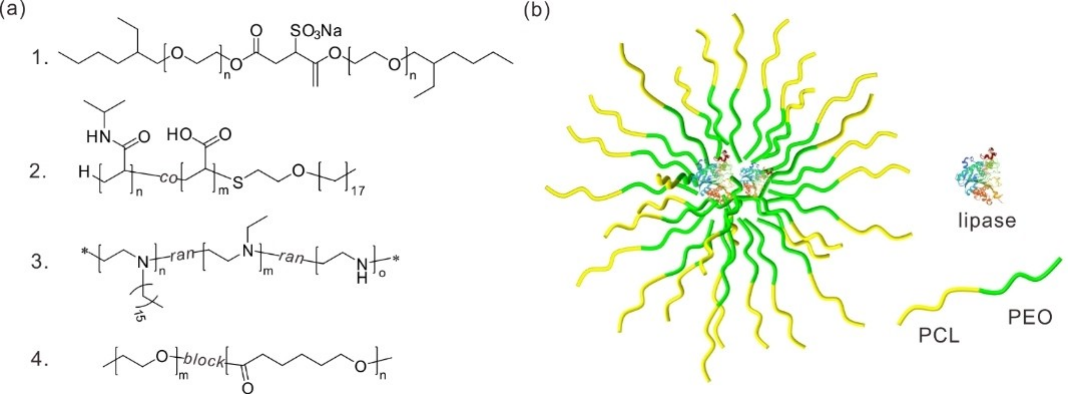
a) Chemical structures of the reported synthetic polymers used in the preparation of reverse micelles for biocatalytic synthesis. b) Schematic depiction of the encapsulation of lipase in reverse micelles self‐assembled from PCL‐*b*‐PEO.

Compared to polymersomes, research on the application of polymeric reverse micelles as biocatalytic reactors remains limited (Figure 5a). Sodium bis(2‐ethylhexyl)sulfosuccinate (AOT) is the most commonly used ionic surfactant for forming reverse micelles. To overcome the negative effect of small molecular AOT on the entrapped biocatalyst in reverse micelles, a polymeric analogue of AOT was developed.^[53]^ This polymeric analogue is sodium bis(2‐ethylhexyl polyoxyethylene)sulfosuccinate (MAOT), which can be structurally viewed as inserting a hydrophilic polyoxyethylene block between the head group and hydrophobic tails of AOT. Reverse micelles formed by MAOT in isooctane showed higher activity and stability than those stabilized by AOT in catalyzing the hydrolysis of entrapped olive oil of *Candida rugosa* lipase. The enzyme *Candida rugosa* lipase was also immobilized in reverse micelles formed by thermoresponsive poly(*N*‐isopropylacrylamide‐*co*‐acrylic acid) (P(NIPAAm‐*co*‐AA)) end‐capped with different alkyl groups.^[54]^ Among the polymers investigated, it was found that undecane end‐capped P(NIPAAm‐*co*‐AA) showed the best performance for immobilizing enzymes. The activity of the entrapped *Candida rugosa* lipase was almost 27 times higher than naked ones dispersing in organic solvent, and its stability was also much higher than that stabilized in AOT micelles. Moreover, the enzyme can be recovered simply by increasing the temperature to a value slightly higher than the lower critical solution temperature (LCST) of the copolymers. Polyethyleneimine (PEI) was modified through an alkylation reaction using cetyl bromide and ethyl bromide, forming a macromolecular surfactant with a hydrophilic backbone and hydrophobic side groups.^[55]^ In the presence of this polymer surfactant, a certain amount of water can be solubilized in benzene/*n*‐butanol mixtures, forming reverse polymeric micelles. A considerable increase in catalytic activity and stability of the entrapped enzyme α‐chymotrypsin was observed as compared to that in an aqueous solution. The Xenakis group reported the use of block amphiphilic copolymers consisting of hydrophilic PEO and hydrophobic poly(ϵ‐caprolactone) (PCL) with different hydrophilic‐to‐hydrophobic ratios.^[56]^ The micellar systems were obtained through self‐assembly of these amphiphilic copolymers in mixtures of chloroform, 2‐propanol, and water, and these systems were then used as bioreactors for a model esterification reaction of 1‐propanol with lauric acid after encapsulating *R. miehei* lipase (Figure 5b). However, degradation of copolymers was observed in this system as the carboxylic group in the copolymers may act as a possible substrate of the encapsulated enzyme, revealing the importance of careful selection of copolymers and biocatalysts when constructing reverse micelle biocatalytic systems. Sodium lignosulfonate vesicular reverse micelles have been shown to immobilize HRP, demonstrating that natural polymers can also be used to form reverse micelles as nanoreactors for biocatalytic synthesis, and offering appealing opportunities to fabricate biocompatible catalytic systems with renewable materials.^[11a]^ The immobilized HRP in these reverse micelle reactors showed a specific activity of 114.41 U mg^−1^, which was more than 5 times higher than that of free HRP at pH 4, indicating the protective role of the reverse micelles against acidic conditions. However, the nonspecific interactions between entrapped HRP and surrounding polymers likely resulted in conformational changes of HRP, and hence the highest specific activity of the entrapped HRP was still lower than that of free HRP.

The great potential of the application of polymeric reverse micelles as nanoscale biocatalytic reactors has been demonstrated, though only a few examples have been reported. In these systems, biocatalysts are positioned in the water core tightly surrounded by amphiphilic polymers, protected from the external organic environment, while large amounts of substrates and products can be well solubilized in the organic solvent. However, deactivation was observed in some cases due to the nonspecific interactions between surrounding amphiphilic polymers and inside biocatalysts. Thus, more and deeper investigations are expected to disclose the origin of this phenomenon and avoid it during catalysis.

## 3 Polymeric Reactors in Biphasic Solutions

In spite of the enormous success of biocatalytic reactors in monophasic solutions, the incompatibility in solubility of biocatalysts and substrates still remains, which severely restricts the range of biocatalytic reactions that can be performed in monophasic conditions. To address this challenge, researchers have developed methods for conducting biocatalytic reactions in biphasic (also called two‐phase) media. A polymeric reactor in biphasic solution typically refers to an emulsion stabilized by amphiphilic polymers or by nanoparticles composed of polymers. In these emulsion systems, a high amount of biocatalysts can be positioned in an aqueous phase while organic substrates are solubilized in an oil phase, solving the incompatibility problem mentioned above. Meanwhile, the mass transfer resistance between the water and oil phases is also significantly reduced in emulsion systems because of their large interfacial area relative to conventional biphasic solutions. Usually, water‐in‐oil (W/O) reverse emulsions are often chosen for biocatalysis, compared to oil‐in‐water (O/W) emulsions, for two reasons: first, the biocatalysts show good performance in confined water environments such as the dispersed phase of emulsions;^[57]^ and second, the dissolution of reactants in the continuous organic phase allows for easy postprocessing.

### 3.1 Polymer Emulsions

In Section 2.1, it is demonstrated that the self‐assembly of amphiphilic copolymers can lead to the formation of polymersomes or reverse micelles that can serve as bioreactors in aqueous or organic solutions, respectively. By contrast, polymer emulsions are generated in a biphasic solution when amphiphilic copolymers self‐assemble at the interface of two phases, resulting in the formation of a compartmentalized structure with micron‐scale diameters.^[58]^ Polymer emulsions combine the advantages of polymersomes and reverse micelles for the construction of biocatalytic nanoreactors. In these emulsions, biocatalysts can be positioned in an aqueous environment, while organic substrates and products are well solubilized in organic phase, solving the incompatibility issues. Our group has conducted a series of studies in this field, and attained some exciting achievements.^[59]^

**Figure 6:**
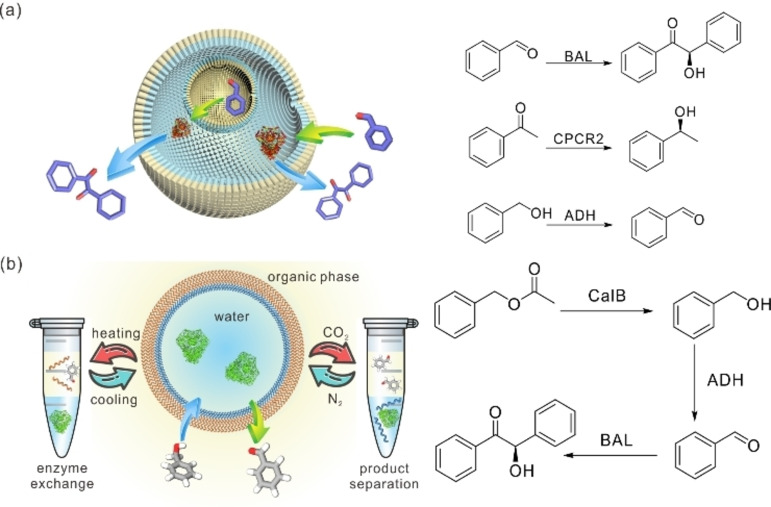
a) Schematic illustration of multi‐compartmentalized polymer emulsions for biocatalytic synthesis, and the reactions catalyzed by different biocatalysts and performed in the multi‐compartmentalized polymer emulsions. The biocatalysts can be free enzymes or enzymes within living cells. Adapted from Ref. [59a] with permission from Wiley‐VCH. Adapted from Ref. [59b] with permission from Elsevier. b) Schematic illustration of the multi‐responsive emulsions stabilized by PDBAEAM‐*b*‐PNIPAAm for biocatalytic synthesis, and the reaction scheme of the three‐step biocatalytic sequential reaction performed in the responsive polymer emulsion. Adapted from Ref. [59d] with permission from Wiley‐VCH.

In our pioneering work about the application of amphiphilic block copolymers for constructing polymeric emulsions for biocatalytic synthesis, stable water‐in‐toluene emulsions were obtained simply by gentle hand‐shaking using the triblock copolymer poly(ethylene glycol)‐*block*‐poly(ϵ‐caprolactone)‐*block*‐poly(ethylene glycol) (PEG‐*b*‐PCL‐*b*‐PEG) as an emulsifier under optimal conditions. It was found that the emulsion structures varied from single‐compartment to multicompartment upon changing the water‐to‐toluene ratio. Due to the mild preparation conditions, high stability, and large interfacial area of the multicompartment emulsion, it was used as a platform for biocatalysis by entrapping vulnerable benzaldehyde lyase (BAL) in the water phase while solubilizing substrates and products in the toluene phase (Figure 6a).^[59a]^ These entrapped BALs were used to catalyze selective carboligation between two benzaldehyde molecules, and the best initial specific activity was 225 times compared to that of BAL in an unemulsified biphasic system. Additionally, the multiple emulsions allowed BAL to maintain 80 % of the initial activity after 24 h of storage, and tolerated a high concentration of substrates. Furthermore, this system can be easily scaled up to at least 2 L for gram‐scale production of expensive enantiopure compounds, showing its potential for large‐scale synthesis. In addition to pure enzymes, we explored the encapsulation of entire cells in this emulsion for biocatalytic reactions.^[59b]^ *Escherichia coli* (*E. coli*) cells overexpressing BAL, *Candida parapsilosis* carbonyl reductase (CPCR2), or thermophilic alcohol dehydrogenase (ADH) were successfully encapsulated in this emulsion system and used for biocatalytic synthesis, indicating the system's universal capability as a biocatalytic reactor. In the case of *E. coli* overexpressing BAL, the multiple W/O emulsion system enhanced the catalytic performance of entire *E. coli* cells by a maximum of 137 times compared to unemulsified biphasic systems. Considering that the cell membrane often presents a severe barrier to substrate and product transport, and that purifying enzymes is usually laborious, time‐consuming, and costly, we combined this multicompartment emulsion with cell‐free protein synthesis (CFPS) technology to generate an artificial enzymatic pathway.^[59c]^ In this biocatalytic system, styrene monooxygenase (SMO) and epoxide hydrolase (SpEH) were generated with the CFPS method in an aqueous phase and applied for a two‐step reaction at the aqueous–organic interface that converted styrene to (*S*)‐1‐phenyl‐1,2‐ethanediol without purification. Due to this reaction's high catalytic efficiency, 100 % conversion of styrene to the target product was achieved even with a concentration below 20 mM. This work presents a new and feasible method for designing efficient biocatalytic reactors for the cost‐effective and high‐yielding synthesis of valuable chemicals. Very recently, our group reported that polymers can function as catalytically active emulsifiers in polymer emulsions for biocatalytic synthesis. In this work, a catalytic polymer obtained from the modification of hyperbranched polyglycerol (hPG) through alkylation and sulfation was applied as both emulsifiers and organocatalysts. This polymer was successfully used to fabricate emulsions for chemoenzymatic cascade reactions with CalB in an aqueous phase catalyzing the hydrolysis of ethylene glycol diacetate to generate ethylene glycol, while the polymer located at the phase interfaces acts as chemical catalysts further catalyzing the acetalization reaction between ethylene glycol and benzaldehyde to produce 2‐phenyl‐1,3‐dioxolane.^[59e]^

Smart emulsions, which can be destabilized on demand, are also of great interest for the easy separation of products and recovery of biocatalysts. In this regard, we developed a type of polymer emulsion that shows multi‐responsive behavior towards CO_2_, pH, and temperature, allowing the on‐demand control of emulsion morphology and phase composition (Figure 6b).^[59d]^ The polymer we used is a diblock copolymer, poly(*N*‐[2‐(dibutylamino)ethyl]acrylamide)‐*b*‐poly(*N*‐isopropylacrylamide) (PDBAEAM‐*b*‐PNIPAAm). The multi‐responsive emulsions allowed not only for single‐step biosynthesis with easy purification, but also for sequential multi‐enzyme cascade reactions. More importantly, the emulsions’ stimuli‐responsiveness allows reaction conditions to be optimally tuned for each step in cascade reactions, simply by pausing the reactions in order to switch reaction media. To illustrate these emulsions’ applicability, we designed a three‐step cascade reaction using three enzymes, CalB, ADH, and BAL, for the production of (*R*)‐benzoin from inexpensive raw materials, specifically benzyl acetate. Due to the optimal reaction conditions and the large interfacial area of the responsive emulsions, a theoretical yield of nearly 97 % was achieved. Moreover, with a reaction volume that can be increased to at least 800 mL, this reaction's scalability represents a significant contribution of polymeric emulsions to the field of biocatalytic synthesis. Considering the diversity of polymer structures, it is easy to envision more smart emulsions being constructed for future enzymatic reactions.

As displayed in this subsection, our group has developed a series of polymer emulsion systems as microreactors for biocatalytic synthesis. In these systems, biocatalysts and substrates are usually positioned in the aqueous and organic phases, respectively. And the large interfacial area between two phases ensures the fast mass transfer, leading to high catalytic activity. But it needs to be noted that the fabricated polymer emulsion systems should have sufficient stability during the catalytic process, which requires careful selection of the amphiphilic polymers and suitable solvents. Besides, it is highly appealing that the polymer emulsions can be obtained under mild conditions. The risk of biocatalyst deactivation during emulsion preparation can be minimized because vigorous shaking or stirring is avoided.

### 3.2 Pickering Emulsions

Pickering emulsions, which are stabilized by nano‐ or microparticles, have been shown to be good substitutes for conventional emulsions stabilized by amphiphilic molecules.^[60]^ As opposed to the dynamic absorption and desorption of amphiphilic molecules at the water/oil interface, in Pickering emulsions, nano‐ or microparticles are irreversibly adsorbed at this interface, and large energy is required to detach them; therefore Pickering emulsions often show much higher stability. Since we reported the first successful encapsulation of lipase in a Pickering emulsion stabilized by SiO_2_ particles,^[61]^ more and more reports have emerged about using Pickering emulsions as bioreactors.[^7b^, ^62^] Polymeric stabilizers, with their diverse range of structures and special properties enabling specific functionalization, have their own advantages over inorganic stabilizers.[^11b^, ^63^] Polymersomes, polymeric nanoparticles, and polymeric microgels are most commonly used to prepare polymeric Pickering emulsions for biocatalytic synthesis.

#### 3.2.1 Pickering Emulsions Stabilized by Colloidosomes

**Figure 7:**
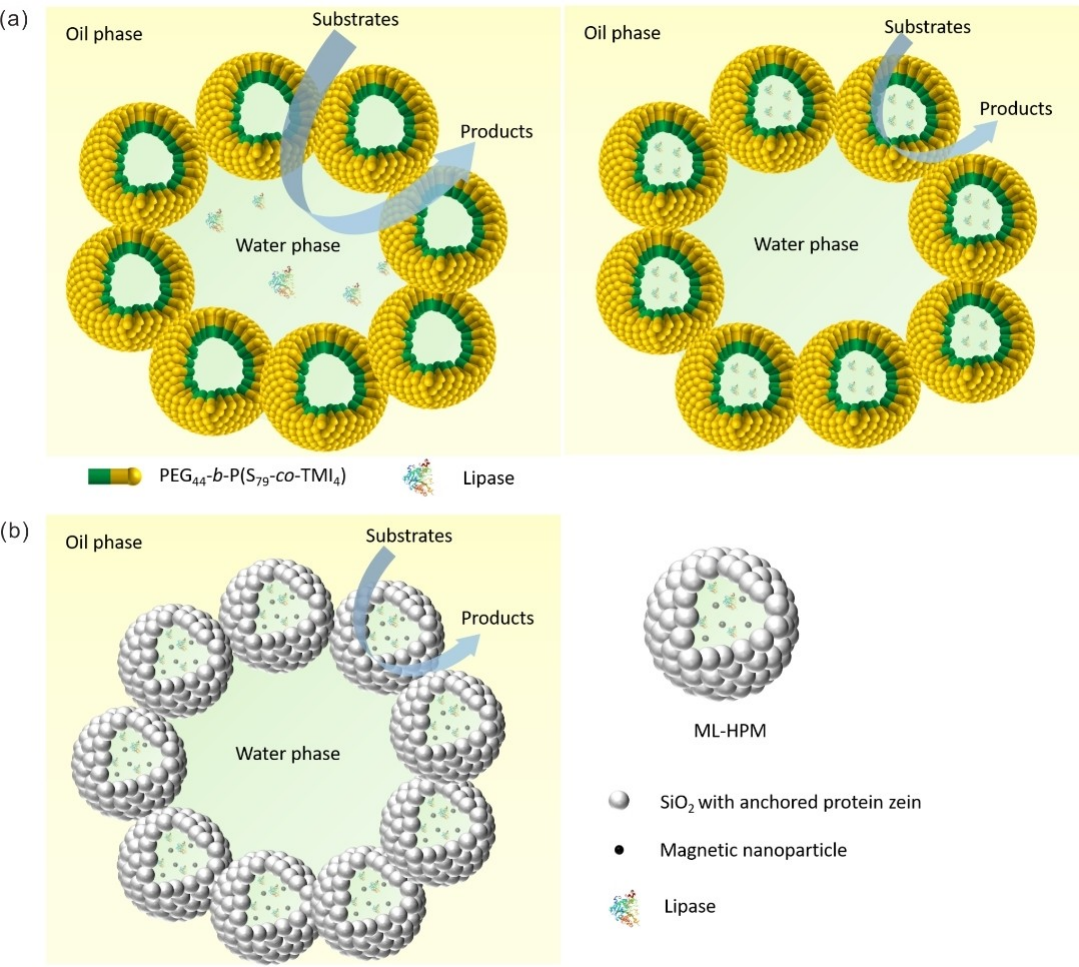
a) Schematic depiction of Pickering emulsions stabilized by polymersomes with enzymes in the water phase and inside the polymersome lumen, respectively. b) Schematic illustration of interfacial catalysis in the Pickering emulsion stabilized by proteinaceous colloidosomes containing lipase in the colloidosome lumen.

As mentioned in Section 2.1, in the context of monophasic systems, polymersomes can encapsulate biocatalysts and serve as microreactors for biocatalysis in an aqueous environment. In a biphasic system, they can also work as so‐called stabilizers of a Pickering emulsion.^[64]^ The first successful construction of an enzyme‐loaded polymersome Pickering emulsion was achieved using poly(ethylene glycol)‐*b*‐poly‐(styrene‐*co*‐3‐isopropenyl‐α,α‐dimethylbenzylisocyanate) (Figure 7a).^[65]^ In this case, CalB was immobilized either in the bulk water phase of the Pickering emulsion system or in the lumen of the polymersomes. The esterification reaction between 1‐hexanol and hexanoic acid was employed as the model reaction. It was found that specific activity of CalB in the lumen of polymersomes (70.8 U mg^−1^) was 2.8 times higher than that of CalB positioned in the water phase (25.2 U mg^−1^). The much higher activity of CalB in the latter case is because the polymersomes containing CalB are located at the interface of water and oil, and so the distance between CalB and substrates is shorter than for CalB solubilized in the bulk water phase. Organic solvents usually have a significant impact on the structural integrity of polymersomes, thus high stability of polymersomes is essential when acting as stabilizers of Pickering emulsions. Castiglione's report described a radical polymerization method with ammonium persulfate (APS) as initiator and tetramethylethylenediamine (TEMED) as accelerator for crosslinking the block copolymer PMOXA_15_‐PDMS_68_‐PMOXA_15_ with terminal methacrylates to enhance the stability of polymersomes.^[66]^ As a result of this method the encapsulated enzyme mandelate racemase (MR) maintained a much higher residual activity compared with that of MR encapsulated in polymersome crosslinked through UV‐initiated polymerization. MR encapsulated in polymersome‐stabilized Pickering emulsion was found to be active for more than 24 h, while free enzyme got completely inactivated within only 1 h, indicating the great potential of such biphasic set‐up for sensitive biocatalysts.

In addition to synthetic polymers, a nontoxic biological polymer, namely the protein zein, was also used to prepare colloidosomes, which were then further used to fabricate Pickering emulsions for biocatalysis (Figure 7b).^[67]^ During preparation of the colloidosomes, hydrophobic silica nanoparticles were introduced to promote their formation. Meanwhile, magnetic nanoparticles (MNPs) and lipase were also incorporated simultaneously into the proteinaceous colloidosomes (ML‐HPM), imparting interfacial catalysis and magnetic response to the obtained water‐in‐oil emulsion. The esterification reaction between 1‐hexanol and hexanoic acid was selected as a model reaction. This proteinaceous colloidosome‐stabilized Pickering interfacial biocatalytic system, with lipase in the colloidosomes, showed enhanced catalytic activity, ease of product separation, and exceptional recyclability as compared to either a conventional biphasic system or a W/O Pickering emulsion with lipase located in the bulk water phase.

#### 3.2.2 Pickering Emulsions Stabilized by Polymeric Nanoparticles

**Figure 8:**
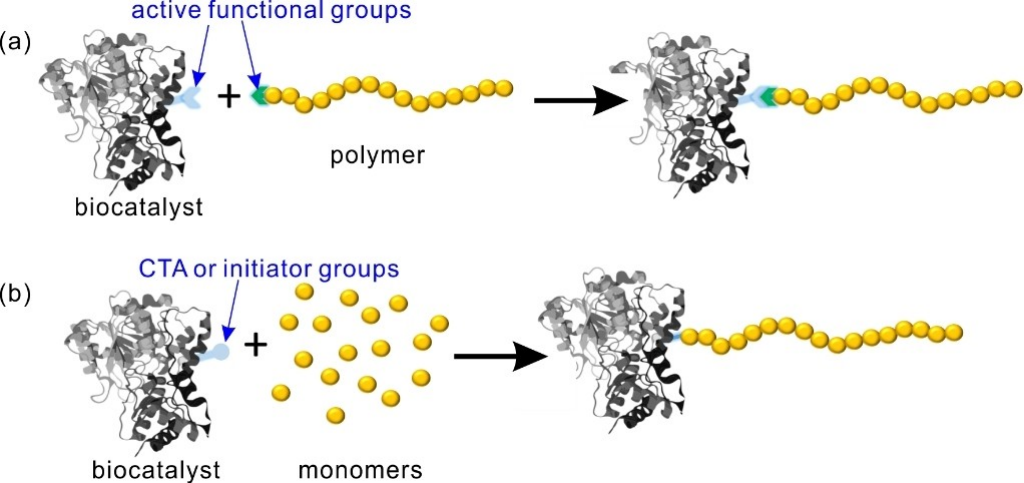
Preparation of polymer–protein conjugates. a) Grafting‐to strategy: polymers are first synthesized and then covalently coupled to proteins. b) Grafting‐from strategy: proteins are modified into macromolecular initiators, and polymerization then takes place *in situ*.

Another popular method of preparing polymeric reverse Pickering emulsions for biocatalysis is the use of polymeric nanoparticles as stabilizers. Polymer–protein conjugates are the most commonly used polymeric nanoparticles for stabilizing Pickering emulsions for biocatalytic synthesis. In these polymer–protein conjugates, the protein can be either the biocatalyst itself or another protein without catalytic ability. Conjugating appropriate polymers to proteins enables the creation of amphiphilic conjugates that can stabilize Pickering emulsions.^[68]^ Meanwhile, the intrinsic characteristics of polymers can also be incorporated into the resulting polymer–protein conjugates.^[69]^ When biocatalysts are used to produce the polymer–protein conjugates, the modification of biocatalysts with polymers can enhance the solubility and stability of biocatalysts in a non‐natural environment.^[70]^ Previous studies also suggest that enzymes located at the interface result in higher efficiency than those located in the bulk water phase.^[65]^ The location of enzymes at the interface can be easily achieved when the Pickering emulsion is stabilized by polymer–biocatalyst conjugates. Methods for preparing polymer–protein conjugates are comprised mostly of so‐called “grafting‐to” and “grafting‐from” strategies (Figure 8).

**Figure 9:**
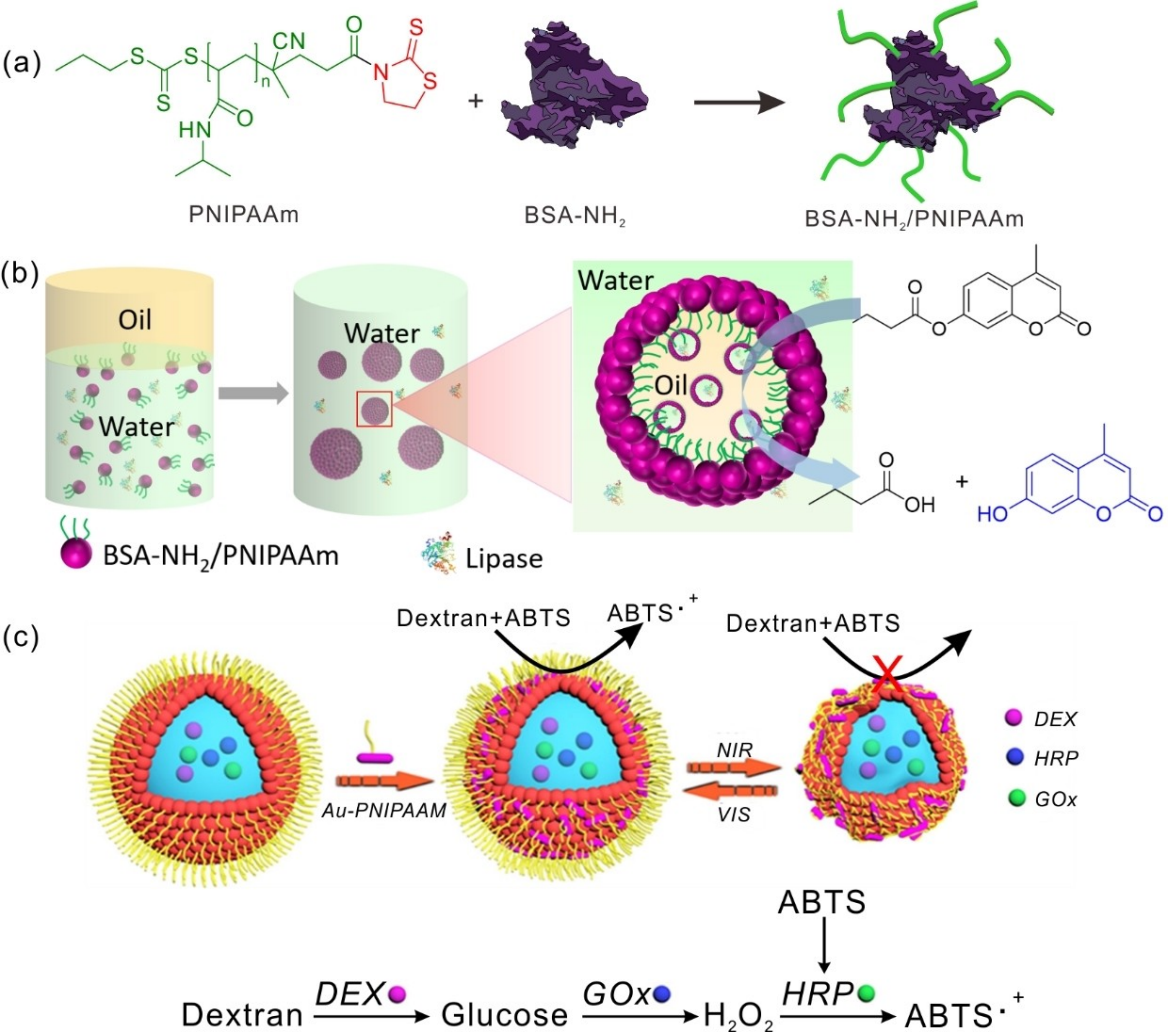
a) Schematic depiction of the preparation of BSA‐NH_2_/PNIPAAm using the grafting‐to strategy. b) Schematic depiction of the preparation of W/O/W Pickering emulsions with BSA‐NH_2_/PNIPAAm conjugates, and their application as a bioreactor for the hydrolysis of MLBB in the presence of lipase. c) Schematic illustration of the biocatalytic cascade reaction carried out in the Pickering emulsions modulated by near‐infrared radiation. Adapted with permission from Ref. [76]. Copyright 2020 American Chemistry Society.

The grafting‐to strategy is a straightforward method for preparing a polymer–protein conjugate usually by coupling polymers directly to proteins via covalent bonds.^[71]^ The biggest advantage of the grafting‐to strategy is the operational ease and precise property control of the polymers, such as narrow polydispersity index (PDI), because the polymerization can be carried out in standard reaction conditions. However, the polymers and proteins may have close molecular weights or strong interactions, and therefore purification after coupling can be a challenge. Moreover, to ensure successful coupling, the polymers are usually used in large excess. One example of the grafting‐to approach is the conjugation of PNIPAAm and bovine serum albumin (BSA) through the reaction of mercaptothiazoline‐activated terminal amides of the PNIPAAm with primary amines on the BSA surface (Figure 9a), as reported by the groups of Mann and Huang.^[72]^ The obtained BSA‐NH_2_/PNIPAAm conjugates are found to self‐assemble at the interface of water and 2‐ethyl‐1‐hexanol to form a Pickering emulsion. The biocatalytic reactor is constructed by dissolving the biocatalyst triglyceride lipase in aqueous phase and the substrate 4‐methyl‐umbelliferyl butyrate (MLBB) in the oil phase during the self‐assembly of BSA‐NH_2_/PNIPAAm conjugates at water/oil interfaces. This reactor's catalytic performance is then investigated by assessing the interfacial lipase‐mediated hydrolysis of MLBB (Figure 9b).^[73]^ A rate enhancement of approximately 11.5 times was observed for the Pickering emulsion system compared with an unemulsified biphasic system. A similar approach is used by the Chen group to prepare mCherry protein–PNIPAAm conjugates, which self‐assemble at the interfaces of water and 2‐ethyl‐1‐hexanol to form a water‐in‐oil Pickering emulsion. This emulsion is successfully applied as a biocatalytic reactor system for a polymerase chain reaction (PCR) to generate DNA upon loading with template DNA, Taq polymerase, primers, and dNTPs in the aqueous phase.^[74]^

Controllable permeability is always an interesting topic when constructing compartmentalized structures for biocatalysis. Huang et al. developed a series of BSA/PNIPAAm conjugates linked via a disulfide bond, which self‐assemble to form multi‐responsive Pickering emulsions.^[75]^ These emulsions’ multi‐responsiveness toward temperature, redox species, and pH allows for the programmable modulation of membrane permeability. Recently, the same group also reported a strategy to fabricate near‐infrared‐responsive Pickering emulsions stabilized by BSA–PNIPAAm conjugates by embedding gold nanorods, which were covalently grafted onto BSA and showed high photothermal conversion efficiency (Figure 9c).^[76]^ Three cascade enzymes, dextran hydrolase (DEX), HRP, and GOx, were encapsulated in the Pickering emulsions to construct a responsive microreactor whose permeability could be controlled due to the compartmentalized structures’ contraction behavior under near‐infrared radiation with the molecular weight cut‐off of the membrane decreasing to ca. 50 kDa. This contraction frequency of the microreactors could be as high as one contraction per minute and last for at least 15 cycles, achieving fast, reversible, and remotely controlled on–off switching of biocatalytic cascade reactions. It is easy to understand that biocatalysts located at the interface of the water and oil phases are beneficial in enhancing catalytic activity because of the enhanced collision efficiency of substrates and biocatalysts; the biocatalysts at interfaces are also protected from being completely exposed to detrimental organic solvents. The Zhang group reported the application of CalB‐decorated polystyrene‐*co*‐poly(glycidyl methacrylate) (P(St‐*co*‐GMA)) nanoparticles as Pickering emulsion stabilizers for biocatalytic reactions. CalB was conjugated onto the P(St‐*co*‐GMA) nanoparticles through reactions between amino and epoxy groups, and localized at the interface of the water and oil phases.^[77]^ This Pickering emulsion system showed a 60.7‐fold improvement in specific activity as compared to an unemulsified biphasic system. Moreover, the CalB–P(St‐*co*‐GMA) nanoparticles could be reused in the Pickering emulsion biocatalytic system over 10 cycles. To facilitate the separation and recycling of biocatalysts, magnetic Fe_3_O_4_ fluid has been introduced during polymerization to form Fe_3_O_4_@PS‐NH_2_ nanoparticles, which can then be further used to prepare Fe_3_O_4_@PS‐NH_2_–lipase conjugates.^[78]^ These conjugates can effectively stabilize the soybean‐oil‐in‐methanol emulsion, and the transesterification reaction showed outstanding enzymatic activity. The highest specific activity of 30.49 U g^−1^ was obtained for the Pickering emulsion system stabilized by polymer–lipase conjugates, which was much higher than that of an unemulsified biphasic system (0 U g^−1^) and Pickering emulsion system containing free lipase (24.92 U g^−1^). Due to the presence of Fe_3_O_4_, Fe_3_O_4_@PS‐NH_2_–lipase conjugates can be easily recovered from the reaction mixture by magnetic attraction and maintained almost constant catalytic activity after being reused for 5 cycles.

**Figure 10:**
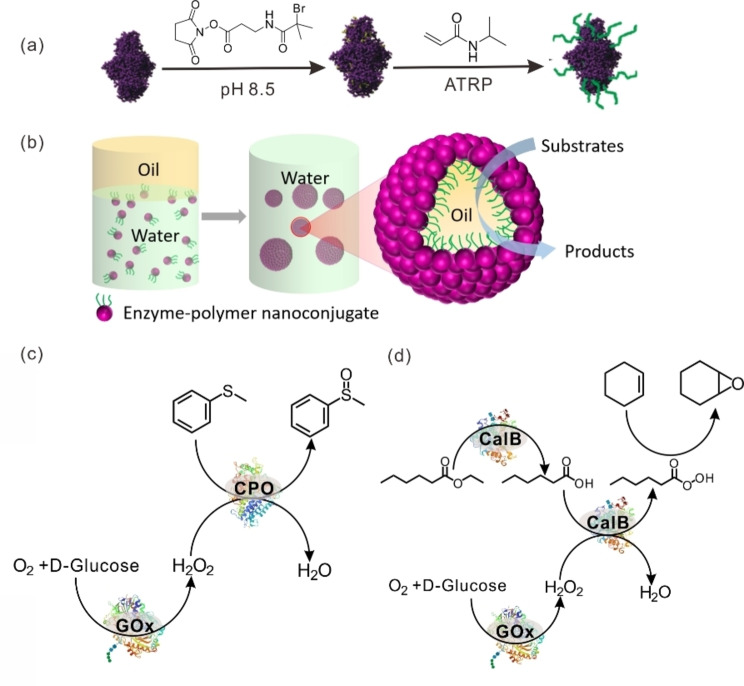
a) Schematic depiction of the preparation of enzyme–PNIPAAm nanoconjugates using the grafting‐from strategy. b) Schematic depiction of the formation of an O/W Pickering emulsion stabilized by enzyme–polymer nanoconjugates. c) Cascade reaction catalyzed by GOx and CPO. d) Cascade reactions catalyzed by GOx and CalB. Adapted from Ref. [80] with permission from Wiley‐VCH.

The grafting‐from strategy to prepare polymer–protein conjugates requires that the polymers grow from proteins. Therefore, the proteins are usually modified to install either a chain transfer agent (CTA) for reversible addition–fragmentation chain‐transfer (RAFT), or an initiator for ATRP before the *in situ* polymerization takes place.^[79]^ In comparison with the grafting‐to strategy, here the polymerization requires only a small excess of monomers, making the grafting‐from method more economical. Additionally, the final synthesized polymer–protein conjugates can be easily separated from non‐reacted monomers and other small‐molecule reagents. However, polymerization in the grafting‐from strategy takes place in an aqueous environment, which is particularly challenging and somewhat limits the scope of monomers. Our group constructed BAL–PNIPAAm conjugates using the grafting‐from strategy by ATRP and subsequently used them to stabilize Pickering emulsions for biocatalysis (Figure 10).^[80]^ Efficient benzoin condensation was achieved in this Pickering emulsion system with a 270‐fold improvement in catalytic performance compared with unemulsified biphasic system. Considering that BAL is a relatively unstable enzyme, this research implies that ATRP is an efficient way to make polymer–enzyme conjugates while maintaining the activity of enzymes. Using a similar method, we further prepared GOx–PNIPAAm conjugates and used them to stabilize a Pickering emulsion. We then successfully performed a three‐step cascade reaction after loading CPO or CalB into this emulsion system, demonstrating the possibility of incorporating a broad spectrum of enzymes and reactions into future synthetic systems.

Distinct from the polymer–protein conjugate nanoparticles mentioned above, Ngai and colleagues recently reported the direct application of phosphorylated zein protein particles as emulsifiers to stabilize Pickering emulsions for biocatalysis.^[81]^ Cascade reactions at the oil–water interfaces were carried out using the zein protein particles with anchored Au nanoparticles and GOx in the aqueous phase. The zein protein particles with anchored Au nanoparticles mimicked certain properties of HRP, which can use H_2_O_2_ from the oxidation of glucose catalyzed by GOx to trigger other reactions. In this case, the reaction system was successfully used to synthesize methyl phenyl sulfoxide. This work highlighted the cooperative application of an artificial enzyme and a bio‐enzyme in a one‐pot cascade Pickering interfacial catalysis.

#### 3.2.3 Pickering Emulsions Stabilized by Microgels

**Figure 11:**
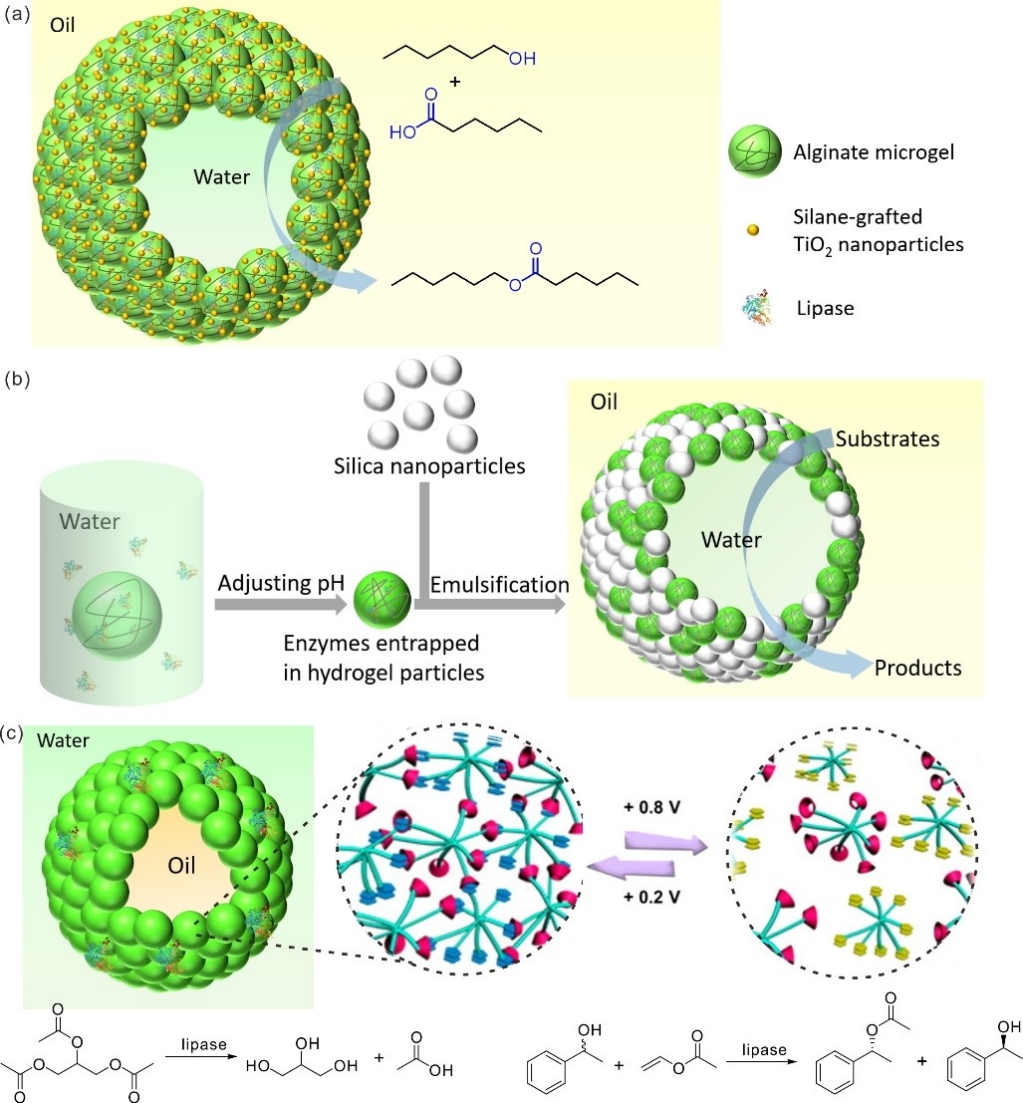
a) Schematic illustration of a Mickering emulsion stabilized by alginate hydrogel particles coated with silane‐grafted TiO_2_ nanoparticles, and its application in a biocatalytic reactor for the esterification of 1‐hexanol and hexanoic acid. b) Schematic illustration of the formation of Mickering emulsions co‐stabilized by PDEAEMA microgel and SiO_2_ nanoparticles for biocatalytic synthesis. c) Schematic illustration of an electrochemically responsive Mickering emulsion, and the catalytic reactions carried out in the Mickering emulsion system containing lipase. Adapted with permission from Ref. [86]. Copyright 2016 American Chemistry Society.

Pickering emulsions stabilized by microgels are also termed Mickering emulsions.^[82]^ These microgels can be prepared from either natural polymers[^11b^, ^83^] or synthetic polymers.^[84]^ Due to their softness, microgels can dramatically swell or shrink in response to external stimuli, allowing for controllable modulation of the permeability and even stability of Mickering emulsions. When Mickering emulsions are used as biocatalytic reactors, biocatalysts can be either solubilized in aqueous solutions or entrapped in microgel particles. The gel particles’ location at the water–oil interface prevents direct contact between biocatalysts and harmful organic solvents, enhancing both catalytic efficiency and the stability of enzymes. Furthermore, phase separation can be achieved by simple centrifugation or filtration, and products can then be extracted from the organic phase, while the microgel particles can be reused for further reactions. Due to their biocompatibility and ease of gelation, alginate hydrogel particles coated with silane‐modified TiO_2_ were used as an emulsifier for Mickering emulsions (Figure 11a).^[85]^ In this Mickering emulsion system, lipase from *Candida* sp. was encapsulated in the alginate microgel particles, which were located at the interface of the water and oil phases. The specific activity of the encapsulated lipase (9.8 U mg^−1^) in catalyzing the esterification of 1‐hexanol and hexanoic acid was 1.4 and 11 times higher than that of free lipase in the water phase of the Pickering emulsion (7.1 U mg^−1^) and a conventional unemulsifier biphasic hexane–water system (0.9 U mg^−1^), respectively. The Lu group designed a core–shell‐structured cellulosic capsule that can stabilize Pickering emulsions for biocatalysis.^[11b]^ This cellulosic capsule had a relatively hydrophobic ethyl cellulose (EC) shell and a hydrophilic interior composed of carboxymethyl cellulose (CMC). The EC shell enabled the capsules to stabilize oil‐in‐water Pickering emulsions, while the water‐rich interior offered a favorable environment for the biocatalyst, which was physically immobilized via hydrogen bonding and electrostatic interactions with the CMC. Esterification between oleic acid and octanol was chosen as the model reaction. It was found that 50.8 % of the reactants were converted after 10 h under room temperature with a small amount of lipase (0.0625 g g^−1^), while only 15.0 % and 24.1 % of the reactants were converted by the same amount of free lipase in an unemulsified biphasic system and a Pickering emulsion system stabilized by empty capsules without lipase inside, respectively. Besides, the water‐rich interior derived from the CMC was also proved to be a key factor in realizing the high catalytic efficiency of encapsulated lipase due to the conformationally free space.

In the case of synthetic polymer microgels, PNIPAAm microgel is a popular choice for constructing Mickering emulsions, but only a few such emulsions are used as biocatalytic reactors.^[84]^ Recently, the Ngai group synthesized a type of pH‐responsive poly(2‐(diethylamino)ethyl methacrylate) (PDEAEMA) microgel that can absorb and confine lipase in response to the pH adjustment and can also stabilize a W/O Mickering emulsion (Figure 11b).^[87]^ With the rigid silica nanoparticles as additional stabilizers, the emulsion structure is remarkably improved, leading to a much higher catalytic efficiency in the esterification reaction of 1‐hexanol with hexanoic acid. Richtering and colleagues developed a series of Pickering emulsions for biocatalysis stabilized by PNIPAAm‐based microgels.^[88]^ The stimuli‐responsive property of PNIPAAm allows for emulsification or phase separation simply by varying temperature or pH, facilitating both the yield of products and the recycling of the microgel. However, in some cases, the application of pH or temperature as a stimulus has its drawbacks, such as the accumulation of salts in the catalytic system, high energy consumption when elevating the temperature, and the denaturation of enzymes under improper pH or high temperature. For these reasons, the search for new catalytic systems responsive to mild stimuli remains attractive but challenging.^[89]^ The Yuan group reported an electrochemically stimulated Mickering emulsion for biocatalysis, which was used for the hydrolysis of triacetin and the kinetic resolution reaction of (*R,S*)‐1‐phenylethanol catalyzed by lipase (Figure 11c).^[86]^ This Mickering emulsion system was stabilized by microgels composed of two kinds of branched copolymers, one containing β‐cyclodextrin (β‐CD) and one containing ferrocene (Fc). The reversible host–guest interactions between β‐CD and Fc endowed the microgels with electrochemical responsiveness because of the redox activity of Fc, leading to the reversible breaking and recovery of the Mickering emulsions.

As a new type of platform for biocatalytic synthesis, Pickering emulsions stabilized by polymeric stabilizers not only possess advantages similar to emulsions stabilized by amphiphilic polymers, but also usually show remarkably high stability. Polymeric colloidosomes, polymeric nanoparticles, and microgel particles have been used as stabilizers in these Pickering emulsions for biocatalytic synthesis. In these systems, enzymes can be located at the water/oil interface for high efficiency of collision with substrates when they are immobilized on or in the emulsion stabilizers. Noteworthily, enzymes are also protected against organic solvents, especially when confined in colloidosomes or hydrogel particles. Besides, these emulsion stabilizers can be also recovered from catalytic systems and reused more easily compared to soluble amphiphilic polymers. However, a precise synthesis procedure is sometimes required to prepare polymeric stabilizers for Pickering emulsions, which poses a challenge to their large‐sale applications.

### 3.3 Static Emulsions

**Figure 12:**
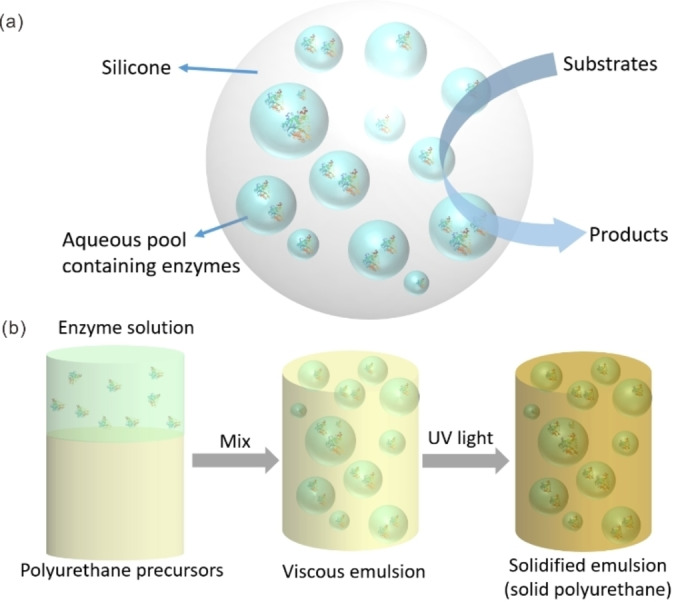
a) Schematic depiction of a silicone‐based static emulsion and its application in biocatalysis. b) Schematic illustration of the preparation of UV‐cured enzyme compartments in a polyurethane‐based static emulsion.

The aforementioned emulsion systems for biocatalysis are fabricated based on the self‐assembly of emulsifiers at the interfaces of two liquid phases. Under unfavorable conditions, such dynamic emulsions still suffer from the issue of instability. In contrast, static emulsion biocatalytic reactors are developed by immobilizing biocatalyst‐containing aqueous solution droplets in solid polymeric matrices, which can preserve a persistent water–organic interface and can also overcome the difficulties in separating products and biocatalysts from reaction systems. The preparation of static emulsions usually involves the formation of a dynamic emulsion through self‐assembly, followed by a subsequent process of solidification. The Ansorge‐Schumacher group pioneered the study of the application of silicone‐elastomer‐based static emulsions for biocatalytic synthesis (Figure 12a).^[90]^ In this work, polymeric ingredients of silicone elastomers were emulsified with an aqueous solution that contained lipase through stirring, during which vulcanization proceeded to generate the static emulsion containing entrapped biocatalysts. These biocatalysts in static emulsions displayed much higher catalytic activity and stability than those entrapped in a sol–gel system, especially after the preparation protocol was optimized.^[91]^ Such static emulsions have been successfully deployed to entrap different lipases, including *Candida Antarctica* lipase A (CalA),^[90]^ *Pseudomonas stutzeri* lipase (lipase TL),^[92]^ lipase of *T. lanuginosa*,^[93]^ lipase from *Burkholderia cepacia*,^[94]^ and hydroxynitrile lyase,^[95]^ indicating their versatility for fabricating biocatalytic systems for a broad range of bioconversions.

Apart from static emulsions based on silicone beads, polyurethane has also been used to construct static emulsions for biocatalytic synthesis, which was reported by von Langermann's group. During the preparation of polyurethane‐based static emulsions, polyurethane precursors were first mixed with aqueous solutions containing biocatalysts to form emulsions, which were then solidified under UV irradiation (Figure 12b).^[96]^ Before use, the solid static emulsion must be cut or ground to the desired size to minimize potential diffusion limitations. As the solidification process only requires UV irradiation treatment under room temperature for about 5 min, relatively unstable enzyme alcohol dehydrogenase from *Lactobacillus kefir* (*Lk*ADH) was successfully encapsulated in the compartments and maintained its catalytic activity comparable to free enzymes in a classical aqueous solution at similar reaction conditions. Additionally, such static emulsions containing separated reaction zones were produced for enzymatic cascade reactions catalyzed by different enzymes requiring contrary reaction conditions. Moreover, the same research group also developed polyurethane‐based static emulsions that entrap entire cells overexpressing alcohol dehydrogenase or esterase for biocatalytic synthesis.^[97]^ This catalysis system shows considerable stability, allowing multiple reuses without a noticeable decline in catalytic activity.

This subsection introduced two families of static emulsions as bioreactors. What they have in common is that emulsion formation must take place before the solidification of the organic phase to generate static emulsions. The solid organic phase should not only provide sufficient mechanical stability for these reactors and protection for encapsulated biocatalysts, but also ensure efficient reactant diffusion through the polymer networks, which necessitate an elaborate design and optimal preparation conditions. Compared to previous emulsion systems, static emulsions are less reported. An important reason is that a relatively harsh preparation condition is often needed, which prohibited many vulnerable enzymes from applications. However, static emulsions are quite easy to prepare, and their outer surfaces are solid‐state, thus making them appealing for downstream processes.

## 4 Summary and Perspectives

The present Review gives an overview of the successes of self‐assembling polymeric nano‐ and microreactors for the application of biocatalysis in organic synthesis, illustrating their structural characteristics and latest advances. The rapid development of polymer science has offered a wide range of polymeric molecules with various building blocks, molecular weights, and hydrophobic or hydrophilic properties, empowering researchers to construct polymeric reactors with a commensurately wide range of different features. Careful selection of biocatalysts, polymers, and strategies for their combination is required, and should be informed by consideration of the synthesis targets and working environments. The self‐assembled biocatalytic polymeric reactors discussed in this Review include polymersomes, reverse micelles, emulsions stabilized by amphiphilic polymers, Pickering emulsions, and static emulsions. Biocatalysts encapsulated in these self‐assembled polymeric reactors are found to show obviously enhanced stability compared to free ones because they are usually positioned in an adaptable environment and protected from harmful factors. Polymersomes and reverse micelles are the representative polymeric reactors for biocatalytic synthesis in aqueous and organic solutions, respectively. Biphasic emulsion systems attract exponentially growing interest because of their high catalytic efficiency derived from high surface areas. The advantages and disadvantages of these polymeric reactors are summarized in Table 1. With the abundant types of polymers and biocatalysts, both small molecules and polymers have been successfully synthesized with these polymeric reactors. Moreover, biocatalytic cascade reaction systems and responsive catalytic systems have also been established. Enzymes and reactions involved in the reported biocatalytic synthesis performed in different self‐assembled polymeric reactors are summarized in Table S1.

**Table 1:** Advantages and disadvantages of different self‐assembled polymeric nano‐ and microreactors for biocatalytic synthesis.

Polymeric reactors		Advantages		Disadvantages
polymersome		aqueous solution as reaction solvent; biocatalysts protected from harmful conditions by polymersome membranes; suppressed cross‐inhibition of enzymes through separating enzymes spatially; improved activity in cascade reactions when biocatalysts are confined in one polymersome		mass transfer limitations across polymer membranes; poor solubility of organic substrates; low entrapment efficiency of biocatalysts in polymersome lumen
polymeric reverse micelle		enzyme is positioned in water core surrounded by polymers; good solubility of organic substrates and products; possibility of superactivity		only a few reported examples; risk of nonspecific interactions between polymers and biocatalysts leading to decreased activity; low loadings of biocatalysts due to the minimal amount of water
polymer emulsion		large interfacial area; high catalytic activity; biocatalysts positioned in aqueous solution with high stability; good solubility of organic substrates and products in organic phase		lower emulsion stability compared with Pickering emulsion and static emulsion systems; risk of biocatalyst inactivation during vigorous agitation when preparing emulsions
Pickering emulsion		large interfacial area; high emulsion stability; biocatalysts can be positioned at the water/oil interface for high frequency of collision with substrates; biocatalysts can be confined within stabilizers for protection; easy recovery and reuse of the particles; good solubility of organic substrates and products in organic phase		complicated synthesis procedures; risk of biocatalyst inactivation during vigorous agitation when preparing emulsions
static emulsion		high mechanical stability; easy to be isolated from reaction media; good solubility of organic substrates and products in reaction media		small interface area; mass transfer limitations; risk of biocatalyst inactivation during formation of emulsions

Despite rapid development and fruitful achievements, it is important to realize that challenges remain. For example, precisely controllable catalytic systems are highly desirable to achieve easy separation, recycling, and continuous processing. This requires that polymeric reactors not only protect and confine biocatalysts but also show responsiveness toward “mild” stimuli rather than changing pH and/or temperature, which can denature vulnerable enzymes. On the other hand, the properties of these self‐assembled polymeric biocatalytic reactors are heavily dependent on the combination of polymers, biocatalysts, and solvents, thus the interactions between them should have a profound impact on their catalytic performances. Yet the influences of these interactions on catalytic performances as well as influencing mechanisms are still poorly understood, which requires further investigations. Furthermore, most investigations into biocatalytic synthesis in polymeric reactors are still proof‐of‐concept studies, with only a few examples demonstrating the application of these catalytic systems for large‐scale synthesis with grams of products. In conclusion, we are still a long way off from transferring these polymeric reactors for biocatalytic synthesis from lab studies to practical applications. General methods for fabricating low‐cost and efficient polymeric biocatalytic reactors on large scale are appealing.

Overall, biocatalysis in polymeric nano‐ and micro‐sized reactors is promising and attractive for organic synthesis. Future development in this area will require the cooperative efforts of biologists, chemists, and researchers from related fields. We anticipate that such fruitful collaboration will result in the emergence of attractive new approaches in this field.

## Conflict of interest

The authors declare no conflict of interest.

5

## Biographical Information

*Yangxin Wang obtained his PhD degree under the supervision of Prof. Ruihu Wang in 2016 at Fujian Institute of Research on the Structure of Matter, Chinese Academy of Sciences. After that, he joined Prof. Zhenhui Qi's group at Northwestern Polytechnical University as a postdoctoral researcher from 2016 to 2019. During this period, he worked at Technische Universität Dresden (TU Dresden) from 2016 to 2018 with Prof. Changzhu Wu, and Karlsruhe Institute of Technology from 2018 to 2019 with Prof. Guillaume Delaittre and Dr. Michael Hirtz as visiting researcher. Since 2020, he has been working as an associate professor at Nanjing Tech University. His current research interests are focused on porous polymeric materials for adsorption and heterogeneous catalysis*.

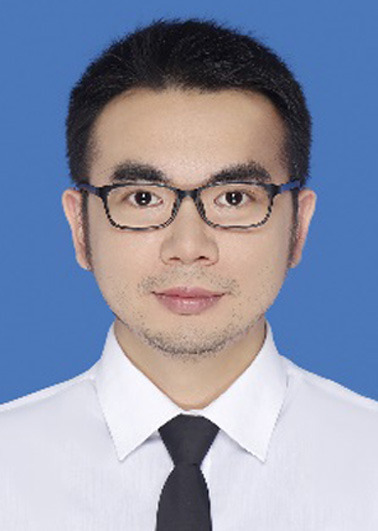

## Biographical Information

*Qingcai Zhao completed an M.Sc. in Polymer Science, which is granted jointly by Freie Universität Berlin (FU Berlin), Humboldt‐Universität zu Berlin, Technische Universität Berlin and University of Potsdam. He then studied macromolecular chemistry in the group of Prof. Rainer Haag at FU Berlin and received his Dr. rer. nat. in 2019. Since 2020 he has been working as research scientist in the FMCG industry*.

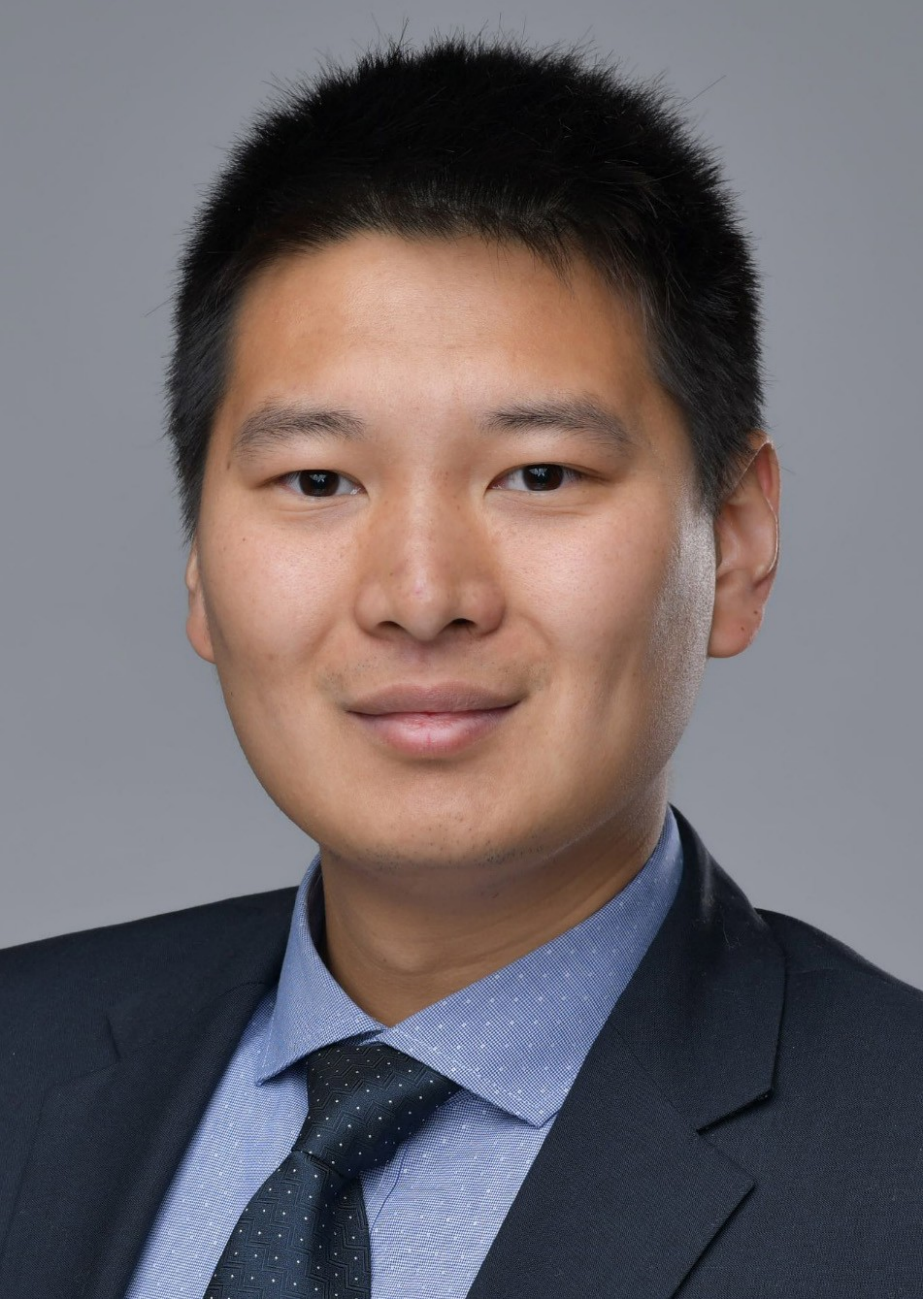

## Biographical Information

*Rainer Haag is Professor of Organic and Macromolecular Chemistry at Freie Universität Berlin. Since 2021, he has been spokesperson of the Collaborative Research Center SFB 1449 “Dynamic Hydrogels at Biological Interfaces”. His research focuses on biodegradable and multivalent macromolecules, supramolecular architectures, nanotransporters for drug delivery, and sustainable polymer syntheses. In start‐up‐oriented teaching, he won the 2014 teaching award at Freie Universität Berlin with his project “Translation of Project Ideas.” Together with the company Dendropharm, he received the Innovation Award Berlin–Brandenburg in 2016. Since 2019, he has been an elected member of the German Academy of Science and Engineering (acatech). In 2022, he was awarded the ERC Advanced Grant*.

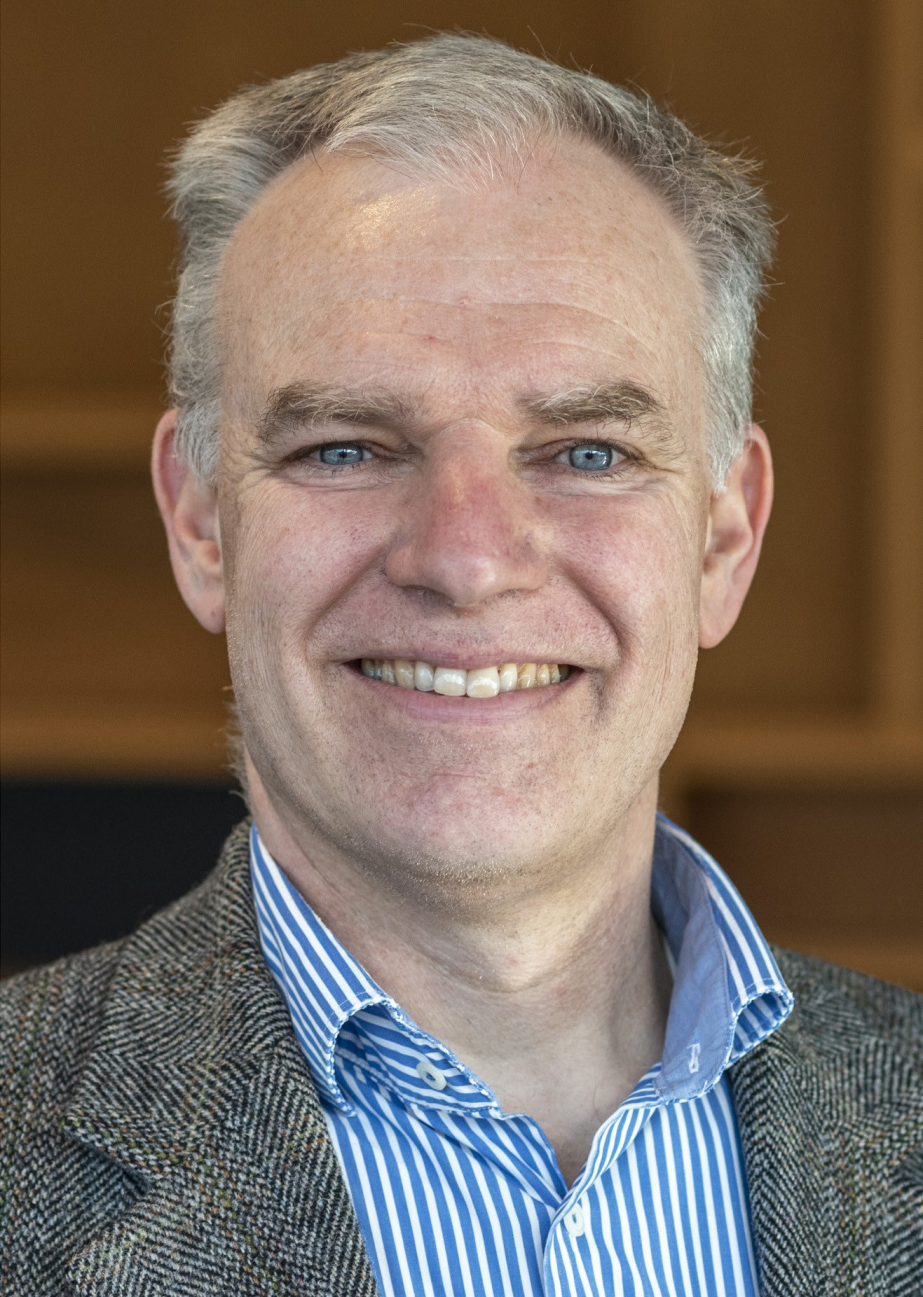

## Biographical Information

*Changzhu Wu is an Associate Professor at the Department of Physics, Chemistry and Pharmacy at the University of Southern Denmark (SDU). His research spans multidisciplinary topics from enzyme catalysis to chemistry and protein chemistry, with a focus on the chemical modification of enzymes, proteins, and cells for catalysis and therapeutics. He had PhD education and Postdoc training at TU Berlin and FU Berlin, respectively. In 2015, he was awarded DFG Temporary Positions for Principal Investigators to start independent research at TU Dresden. In 2018, he received a Professorship call to establish a chemistry and biotechnology research team at SDU, and a year later, he was awarded the prestigious “DFF Sapere Aude Research Leader” grant. For more information, see: www.wugroup.sdu.dk*.

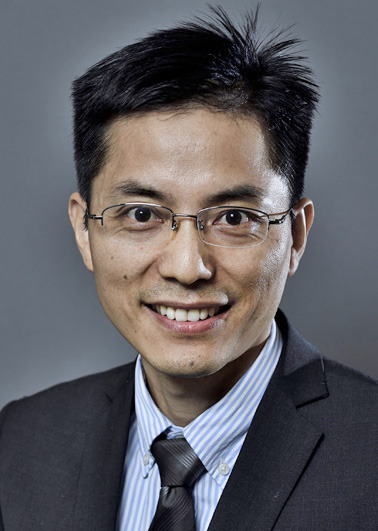

## Supporting information

As a service to our authors and readers, this journal provides supporting information supplied by the authors. Such materials are peer reviewed and may be re‐organized for online delivery, but are not copy‐edited or typeset. Technical support issues arising from supporting information (other than missing files) should be addressed to the authors.

Supporting Information

## Data Availability

Data sharing is not applicable to this article as no new data were created or analyzed in this study.
