# Supplementary material for: Biocatalytic Synthesis Using Self‐Assembled Polymeric Nano‐ and Microreactors
Source: Angew Chem Int Ed Engl. 2022 Nov 17;61(52):e202213974. doi: 10.1002/anie.202213974 (PMC10100074; doi:10.1002/anie.202213974)
Supplement: Supplementary file 1 — Supporting Information [file ANIE-61-0-s001.pdf]

Angewandte Chemie
Eine Zeitschrift der Gesellschaft Deutscher Chemiker

Supporting Information

Biocatalytic Synthesis Using Self-Assembled Polymeric Nano- and Microreactors

*Y. Wang, Q. Zhao, R. Haag, C. Wu**

Table S1. Summary of the reported biocatalytic synthesis in self-assembled polymeric reactors

Enzymes		Reactions	References about different self-assembled polymeric reactors				
			Polymer-somes	Reverse micelles	Polymer emulsions	Pickering emulsions	Static emulsions
Peroxidase	Horseradish Peroxidase (HRP)	Oxidation	[1], [2], [3], [4], [5], [6], [7], [8]	[9]	--	[10]	--
		Radical polymerization	[11], [12]	--	--	--	--
	Chloroperoxidase (CPO)	Oxidation	[13], [14]	--	--	[15]	--
Dehydroge-nase and reductase	Alcohol dehydrogenase (ADH)	Alcohol oxidation	[16]	--	[17], [18]	--	--
		Ketone reduction	--	--	--	[19]	[20], [21]
	Glucose dehydrogenase	Glucose oxidation	--	--	--	--	[20]
	Candia parapsilosis carbonyl reductase (CPCR2)	Carbonyl reduction	--	--	[17]	--	--
Esterase and lipase		Hydrolysis	[6], [7], [13], [16], [22], [23]	[24]	[18],[25]	[15], [26], [27]	[21]
		Transesterification	--	--	--	[28], [29]	[30], [31]
		Esterification	--	[32], [33]	--	[34], [35], [36], [37], [38], [39]	[40], [41]
		Oxidation	--	--	--	[15]	--
		Kinetic resolution	[42]	--	--	[27]	[43]
Oxidase	Glucose oxidase (GOx)	Oxidation	[3], [4], [6], [7], [13]	--	--	[10], [15]	--

	Baeyer-Villiger monooxygenase (PAMO)	Oxidation	[16]	--	--	--	--
	Uricase (UOX)	Oxidation	[8]	--	--	--	--
	Styrene monooxygenase (SMO)	Styrene oxidation	--	--	[44]	--	--
	Laccase	Oxidation	[1], [45]	[46]	--	--	--
Amidase	Penicillin acylase	Penicillin synthesis	[47]	--	--	--	--
	Trypsin	Amide hydrolysis	[48]	[46]	--	--	--
	Alcalase	Hydrolysis	[16]	--	--	--	--
	L-asparaginase (L-ASNase)	L-Asn hydrolysis	[49]	--	--	--	--
Hydrolase	Organophosphorus hydrolase (OPH)	Hydrolysis of organophosphate	--	--	--	[50]	--
	Urease	Hydrolysis of urea	--	--	--	[51]	--
	Epoxide hydrolase (SpEH)	Hydrolysis of epoxide	--	--	[44]	--	--
	Dextran hydrolase (DEX)	Hydrolysis of Dextran	--	--	--	[10]	--
Lyase	N-acetylneuraminate lyase (NAL)	Aldol condensation	[52]	--	--	--	--
	Benzaldehyde lyase (BAL)	Benzoin formation	--	--	[18], [53],	[15]	--
	Hydroxynitrile lyase	Acetone cyanohydrin formation	--	--	--	--	[20], [54]
N-acyl- D-glucosamine 2-epimerase (AGE),		Isomerization	[52]	--	--	--	--

CMP-sialic acid synthetase	Phosphate formation	[52]	--	--	--	--
Mandelate racemase	Racemization	[42]	--	--	[55]	--
Taq polymerase	Polymerase chain reaction (PCR)	--	--	--	[56]	--
Myoglobin	oxidation	[57]	--	--	--	--

References

[1] M. V. Dinu, I. A. Dinu, S. S. Saxer, W. Meier, U. Pieles, N. Bruns, *Biomacromolecules* **2021**, *22*, 134-145.
[2] M. Spulber, A. Najer, K. Winkelbach, O. Glaied, M. Waser, U. Pieles, W. Meier, N. Bruns, *J. Am. Chem. Soc.* **2013**, *135*, 9204-9212.
[3] W. Siti, H. M. de Hoog, O. Fischer, W. Y. Shan, N. Tomczak, M. Nallani, B. Liedberg, *J. Mater. Chem. B* **2014**, *2*, 2733-2737.
[4] D. Grafe, J. Gaitzsch, D. Appelhans, B. Voit, *Nanoscale* **2014**, *6*, 10752-10761.
[5] C. Edlinger, T. Einfalt, M. Spulber, A. Car, W. Meier, C. G. Palivan, *Nano Lett.* **2017**, *17*, 5790-5798.
[6] S. F. van Dongen, M. Nallani, J. J. Cornelissen, R. J. Nolte, J. C. van Hest, *Chem. Eur. J.* **2009**, *15*, 1107-1114.
[7] D. M. Vriezema, P. M. Garcia, N. Sancho Oltra, N. S. Hatzakis, S. M. Kuiper, R. J. Nolte, A. E. Rowan, J. C. van Hest, *Angew. Chem. Int. Ed.* **2007**, *46*, 7378-7382.
[8] A. Belluati, I. Craciun, J. Liu, C. G. Palivan, *Biomacromolecules* **2018**, *19*, 4023-4033.
[9] X. Zhong, Y. Qian, J. Huang, D. Yang, Y. Deng, X. Qiu, *Ind. Eng. Chem. Res.* **2016**, *55*, 2731-2737.
[10] J. Su, H. Chen, Z. Xu, S. Wang, X. Liu, L. Wang, X. Huang, *ACS Appl. Mater. Interfaces* **2020**, *12*, 41079-41087.
[11] M. V. Dinu, M. Spulber, K. Renggli, D. Wu, C. A. Monnier, A. Petri-Fink, N. Bruns, *Macromol. Rapid Commun.* **2015**, *36*, 507-514.
[12] O. Rifaie-Graham, N. F. B. Galensowske, C. Dean, J. Pollard, S. Balog, M. G. Gouveia, M. Chami, A. Vian, E. Amstad, M. Lattuada, N. Bruns, *Angew. Chem. Int. Ed.* **2021**, *60*, 904-909.
[13] H. M. De Hoog, I. W. Arends, A. E. Rowan, J. J. Cornelissen, R. J. Nolte, *Nanoscale* **2010**, *2*, 709-716.
[14] H. M. de Hoog, M. Nallani, J. J. Cornelissen, A. E. Rowan, R. J. Nolte, I. W. Arends, *Org. Biomol. Chem.* **2009**, *7*, 4604-4610.
[15] Z. Sun, U. Glebe, H. Charan, A. Boeker, C. Wu, *Angew. Chem. Int. Ed.* **2018**, *57*, 13810-13814.
[16] R. J. Peters, M. Marguet, S. Marais, M. W. Fraaije, J. C. van Hest, S. Lecommandoux, *Angew. Chem. Int. Ed.* **2014**, *53*, 146-150.
[17] Q. Zhao, M. B. Ansorge-Schumacher, R. Haag, C. Wu, *Bioresour. Technol.* **2020**, *295*, 122221.
[18] Z. Y. Sun, Q. C. Zhao, R. Haag, C. Z. Wu, *Angewandte Chemie-International Edition*, **2021**, *60,* 8410-8414.
[19] S. Wiese, A. C. Spiess, W. Richtering, *Angew. Chem. Int. Ed.* **2013**, *52*, 576-579.
[20] D. Uhrich, J. von Langermann, *Front. Microbiol.* **2017**, *8*, 2111.
[21] D. Uhrich, H. Y. Jang, J. B. Park, J. von Langermann, *J. Biotechnol.* **2019**, *289*, 31-38.
[22] K. T. Kim, J. J. L. M. Cornelissen, R. J. M. Nolte, J. C. M. van Hest, *Adv. Mater.* **2009**, *21*, 2787-2791.
[23] M. Nallani, H. Hoog, J. J. Cornelissen, A. Palmans, J. C. van Hest, R. J. Nolte, *Biomacromolecules* **2007**, *8*, 3723-3728.
[24] J. C. Wu, Z. M. He, C. Y. Yao, K. T. Yu, *J. Chem. Technol. Biotechnol.* **2001**, *76*, 949-953.
[25] Z. Sun, Q. Zhao, R. Haag, C. Wu, *ChemCatChem* **2022**, *14*, e202101556.
[26] L. Wang, P. Wen, X. Liu, Y. Zhou, M. Li, Y. Huang, L. Geng, S. Mann, X. Huang, *Chem. Commun.* **2017**, *53*, 8537-8540.
[27] L. Peng, A. Feng, S. Liu, M. Huo, T. Fang, K. Wang, Y. Wei, X. Wang, J. Yuan, *ACS Appl. Mater. Interfaces* **2016**, *8*, 29203-29207.
[28] L. Qi, Z. G. Luo, X. X. Lu, *Green Chem.* **2019**, *21*, 2412-2427.
[29] X. Liu, Y. Mao, S. Yu, H. Zhang, K. Hu, L. Zhu, J. Ji, J. Wang, *Green Chem.* **2021**, *23*, 966-972.
[30] M. Schlepütz, A. Buthe, R. Brenneis, M. B. Ansorge-Schumacher, *Biocatal. Biotransform.* **2009**, *26*, 220-227.
[31] L. Ma, L. Zhou, Y. Jiang, Y. He, L. Wang, J. Gao, *J. Chem. Technol. Biotechnol.* **2017**, *92*, 1248-1255.
[32] H. Chen, L. H. Liu, L. S. Wang, C. B. Ching, H. W. Yu, Y. Y. Yang, *Adv. Funct. Mater.* **2008**, *18*, 95-102.
[33] V. Sereti, M. Zoumpanioti, V. Papadimitriou, S. Pispas, A. Xenakis, *J. Phys. Chem. B* **2014**, *118*, 9808-9816.
[34] Z. Wang, M. C. van Oers, F. P. Rutjes, J. C. van Hest, *Angew. Chem. Int. Ed.* **2012**, *51*, 10746-10750.
[35] H. Jiang, X. Hu, Y. Li, C. Yang, T. Ngai, *Chem. Sci.* **2021,** *12*, 12463-12467.
[36] M. Wang, M. Wang, S. Zhang, J. Chen, *React. Chem. Eng.* **2019**, *4*, 1459-1465.
[37] X. He, B. P. Binks, J. G. Hu, I. Gates, Q. Y. Lu, *Langmuir* **2021**, *37*, 810-819.
[38] X. Yang, Y. L. Wang, R. X. Bai, H. L. Ma, W. H. Wang, H. J. Sun, Y. M. Dong, F. M. Qu, A. M. Tang, T. Guo, B. P. Binks, T. Meng, *Green Chem.* **2019**, *21*, 2229-2233.
[39] H. Jiang, L. D. Liu, Y. X. Li, S. W. Yin, T. Ngai, *ACS Appl. Mater. Interfaces* **2020**, *12*, 4989-4997.
[40] A. Buthe, A. Kapitain, W. Hartmeier, M. B. Ansorge-Schumacher, *J. Mol. Catal. B: Enzym.* **2005**, *35*, 93-99.
[41] C. Wu, M. Kraume, M. B. Ansorge-Schumacher, *ChemCatChem* **2011**, *3*, 1314-1319.
[42] F. Golombek, M. Haumann, M. S. G. Knoll, A. P. Froba, K. Castiglione, *ACS Omega* **2021**, *6*, 29192-29200.
[43] P. Hoyos, A. Buthe, M. B. Ansorge-Schumacher, J. V. Sinisterra, A. R. Alcántara, *J. Mol. Catal. B: Enzym.* **2008**, *52-53*, 133-139.
[44] W. Q. Liu, C. Wu, M. C. Jewett, J. Li, *Biotechnol. Bioeng.* **2020**, *117*, 4001-4008.
[45] M. Spulber, P. Baumann, S. S. Saxer, U. Pieles, W. Meier, N. Bruns, *Biomacromolecules* **2014**, *15*, 1469-1475.
[46] Y. Khmelnitsky, A. Gladilin, V. Roubailo, K. Martinek, A. Levashov, *Eur. J. Biochem.* **1992**, *206*, 737-745.
[47] K. Langowska, C. G. Palivan, W. Meier, *Chem. Commun.* **2013**, *49*, 128-130.
[48] L. Messager, J. R. Burns, J. Kim, D. Cecchin, J. Hindley, A. L. Pyne, J. Gaitzsch, G. Battaglia, S. Howorka, *Angew. Chem. Int. Ed.* **2016**, *55*, 11106-11109.
[49] J. Qiao, J. Jiang, L. Liu, J. Shen, L. Qi, *ACS Appl. Mater. Interfaces* **2019**, *11*, 15133-15140.
[50] N. Suthiwangcharoen, R. Nagarajan, *Biomacromolecules* **2014**, *15*, 1142-1152.
[51] L.-H. Xue, C.-Y. Xie, S.-X. Meng, R.-X. Bai, X. Yang, Y. Wang, S. Wang, B. P. Binks, T. Guo, T. Meng, *ACS Macro Lett.* **2017**, *6*, 679-683.

[52] L. Klermund, S. T. Poschenrieder, K. Castiglione, *ACS Catal.* **2017**, *7*, 3900-3904.
[53] Q. C. Zhao, M. B. Ansorge-Schumacher, R. Haag, C. Z. Wu, *Chem. Eur. J.* **2018**, *24*, 10966-10970.
[54] J. von Langermann, S. Wapenhensch, *Adv.Synth. Catal.* **2014**, *356*, 2989-2997.
[55] F. Golombek, K. Castiglione, *Biotechnol. J.* **2020**, *15*, e1900561.
[56] X. Wang, Y. Liu, J. Liu, Z. Chen, *ChemBioChem* **2018**, *19*, 1044-1048.
[57] J. Gaitzsch, D. Appelhans, L. Wang, G. Battaglia, B. Voit, *Angew. Chem. Int. Ed.* **2012**, *51*, 4448-4451.
